# Identification of QTLs for Resistance to Sclerotinia Stem Rot and *BnaC.IGMT5.a* as a Candidate Gene of the Major Resistant QTL *SRC6* in *Brassica napus*

**DOI:** 10.1371/journal.pone.0067740

**Published:** 2013-07-02

**Authors:** Jian Wu, Guangqin Cai, Jiangying Tu, Lixia Li, Sheng Liu, Xinping Luo, Lipeng Zhou, Chuchuan Fan, Yongming Zhou

**Affiliations:** National Key Laboratory of Crop Genetic Improvement, Huazhong Agricultural University, Wuhan, China; New Mexico State University, United States of America

## Abstract

Stem rot caused by *Sclerotinia sclerotiorum* in many important dicotyledonous crops, including oilseed rape (*Brassica napus*), is one of the most devastating fungal diseases and imposes huge yield loss each year worldwide. Currently, breeding for *Sclerotinia* resistance in *B. napus*, as in other crops, can only rely on germplasms with quantitative resistance genes. Thus, the identification of quantitative trait locus (QTL) for *S. sclerotiorum* resistance/tolerance in this crop holds immediate promise for the genetic improvement of the disease resistance. In this study, ten QTLs for stem resistance (SR) at the mature plant stage and three QTLs for leaf resistance (LR) at the seedling stage in multiple environments were mapped on nine linkage groups (LGs) of a whole genome map for *B. napus* constructed with SSR markers. Two major QTLs, *LRA9* on LG A9 and *SRC6* on LG C6, were repeatedly detected across all environments and explained 8.54–15.86% and 29.01%–32.61% of the phenotypic variations, respectively. Genotypes containing resistant *SRC6* or *LRA9* allele showed a significant reduction in disease lesion after pathogen infection. Comparative mapping with Arabidopsis and data mining from previous gene profiling experiments identified that the Arabidopsis homologous gene of *IGMT5* (At1g76790) was related to the *SRC6* locus. Four copies of the *IGMT5* gene in *B. napus* were isolated through homologous cloning, among which, only *BnaC.IGMT5.a* showed a polymorphism between parental lines and can be associated with the *SRC6*. Furthermore, two parental lines exhibited a differential expression pattern of the *BnaC.IGMT5.a* gene in responding to pathogen inoculation. Thus, our data suggested that *BnaC.IGMT5.a* was very likely a candidate gene of this major resistance QTL.

## Introduction

*Sclerotinia sclerotiorum* (Lib.) de Bary is a necrotrophic and non-host-specific fungal pathogen that infects more than 400 plant species, including several important oil crops such as oilseed rape, soybean and sunflower [1], [2]. Stem rot in oilseed rape (*Brassica napus*) caused by *S. sclerotiorum* is one of the most devastating diseases for the important crop worldwide. It imposes, for example, 10–20% of yield losses every year, and up to 80% in severely infected fields in China [3], one of the largest rapeseed producers in the world (FAOSTAT data 2011, http://faostat.fao.org/site/567/default.aspx#ancor). In addition to severe seed yield damage, the disease also causes reduction of oil content and changes in fatty acid profile, thus resulting in inferior quality in rapeseeds [4].

The stem rot fungus produces sclerotia, which is a long-lived melanized resting structure [5]. Sclerotia geminate under favorable conditions through either formation of apothecia that release ascospores, or of mycelia that generate hyphae. This life cycle pattern, together with its wide host range, makes Sclerotinia stem rot in oilseed rape difficult to manage through cultural practices. Although several fungicides are available for the control of the disease, it is difficult to pinpoint the optimal time to apply these fungicides, thus often resulting in ineffective sprays [6]. Furthermore, the use of fungicides may cause environmental contaminations and increases farming costs. Therefore, breeding and cultivation of resistant varieties is the most efficient, economic and environment-friendly approach to the controlling of Sclerotinia stem rot. However, breeding of *Sclerotinia*-resistant varieties is confronted with two major difficulties at present. First, no immune or highly resistant germplasm in *B. napus* and its close relatives has been reported so far [7], [8]. Second, the molecular mechanism of the interaction of pathogenic infection and resistance reaction in host plants is poorly understood [5].

Over the last three decades, resistance/tolerance variations of Sclerotinia stem rot in *B. napus* and its close relative species have been investigated and germplasms with partial resistance to Sclerotinia stem rot have been used in *Sclerotinia* resistance breeding. Cultivars with improved resistance to *S. sclerotiorum*, such as ZhongYou 821 [9] and Zhongshuang 9 [10] have been developed and genetic analyses of *Sclerotinia* resistance have been carried out using these materials in *B. nap*us. Classic genetic studies have shown that the resistance to *S. sclerotiorum* in *B. napus* was mainly controlled by quantitative genes with additive effects [11]–[14]. In recent years, mapping of the quantitative trait locus (QTL) for resistance to *S. sclerotiorum* has been conducted in *B. napus*. Three QTLs (located on the linkage group (LG) 3, LG12 and LG17, respectively) involved in leaf resistance at the seedling stage and three different QTLs (LG7, LG10 and LG15) for stem resistance at the mature plant stage in an F_2∶3_ population were identified, and no common QTL was associated with both measures in leaves and stems [15]. Using two doubled haploid (DH) populations (HUA and MS), Zhao et al. (2006) mapped eight QTLs (located on N2, N3, N5, N12, N14, N16 and N19) and one (N3) for the resistance to *S. sclerotiorum* [8]. Yin et al. (2010) identified ten, one and ten QTLs corresponding to three different inoculation methods in a DH population consisting of 72 lines [7]. So far, all the QTLs identified for *Sclerotinia* resistance only explained a small portion of the phenotypic variation. Few QTLs could be detected repeatedly in different populations, environments or with inoculation methods.

Gene expression changes associated with *S. sclerotiorum* infection have been investigated using microarray with different inoculation tissues including leaf [16], petiole [17] and stem [18]. These studies have revealed that several subsets of genes were differentially regulated after infection, such as defense-related genes, phytohormone-responsive genes, transcription factors, as well as genes involved in secondary metabolism and cell wall structure [17], [18]. A large part of these genes exhibits temporal and quantitative differences between resistant genotypes and susceptible ones [17], [18]. However, the relationships of the identified QTLs and the genes responding to pathogen inoculation in gene expression analysis are largely unknown.

With the long-term goal to develop an effective strategy for genetic improvement of the resistance to *S. sclerotiorum*, the current study was aimed to identify major QTLs for Scleratinia stem rot in *B. napus*, which can be immediately used in disease resistance breeding. Two widely used disease assay procedures, detached leaf inoculation [15], [19] and stem inoculation [15], [20] with mycelial agar plugs, were employed to map QTLs for leaf resistance (LR) at the seedling stage and stem resistance (SR) at the mature plant stage in different environments using a DH population. A major QTL (*LRA9*) for LR and a major QTL (*SRC6)* for SR were identified. Through comparative mapping, extensive data mining and homologous cloning, we identified *BnaC.IGMT5.a* as the candidate gene for *SRC6*.

## Materials and Methods

### Plant Materials

A DH population consists of 190 individual DH lines named as the HJ-DH population was used for trait analyses and genetic mapping in this study. The population was developed from microspore culture of F1 buds of the cross between *B. napus* genotypes, Huashuang 5 (Hua 5 thereafter), a cultivar with low *Sclerotinia* susceptibility (susceptible parent), and J7005, a pure line with partly *Sclerotinia* resistance (resistant parent).

### Field Experiments

The HJ-DH lines, along with their parental lines, were grown in disease nursery plots located at the experimental farm of Huazhong Agricultural University, Wuhan, China in three consecutive growing seasons from 2009 to 2012. The field trial did not require any specific permits as the nursery was set up for this type study. Experiments for stem resistant assay at the mature plant stage were performed in the seasons of 2009–2010 and 2010–2011, while for leaf resistant assay in 2010–2011 and 2011–2012. Furthermore, a same set of DH lines was grown for stem resistant assay in the experimental farm of Huanggang Academy of Agricultural Sciences, Huanggang, China in the season of 2010–2011. The field experiment was granted permission by the administrative board of the Huanggang Academy of Agricultural Sciences. Apart from the season of 2009–2010 when 71 lines out of the 190 DH lines were randomly sampled and grown for an initial analysis of stem resistance at the mature plant stage, 190 DH lines were used for all the environments and growing seasons. All the field trials in this study did not involve endangered or protected species.

All the field experiments followed a randomized complete block design with three replications. Each line was planted in one row of 12 plants, with a distance of 21 cm between plants within each row and 30 cm between rows. The field management followed essentially regular breeding practice. Dates to bolting, budding and flowering in the DH population were recorded throughout the whole growth and development period in Wuhan, 2010–2011.

### Assessment of Resistance to *S. sclerotiorum*

An isolate of *S. sclerotiorum*, SS-1, kindly provided by Dr. Guoqing Li from the State Key Laboratory of Agricultural Microbiology, Huazhong Agricultural University, was maintained and cultured on potato dextrose agar (PDA, 25% potato, 2.5% dextrose and 1.5% agar, pH 5.8). The isolate was maintained at 4°C in darkness, and cultured twice before inoculation at 23°C in darkness. Mycelial agar plug (7 mm in diameter) punched from the margin of a 2-day-old culture of *S. sclerotiorum* grown on PDA were used as the inoculum [15], [21].

Two inoculation procedures as described in [15], [20] with minor modifications were used for assessing the resistance to *S. sclerotiorum*. The first one dealt with detached leaves inoculation with mycelial agar plugs in growth room for evaluating leaf resistance (LR) at the seedling stage. The latest or penultimate fully extended leaves with similar size were excised from six- or eight-week old plants grown in the field (November and December of 2010 and 2011). Eight leaves of each DH line were collected and placed in a plastic tray (56 cm×38 cm×15 cm) with wet-gauze at the bottom of the tray. The mycelial agar plug was inoculated on the middle of each leaf. The inoculated leaves were sprayed with a fine mist of water, and the plastic tray was covered with plastic film to maintain a high level of relative humidity. The plastic trays with inoculated leaves were kept at 22±2°C in darkness. The lesion sizes (S) was measured two days post inoculation (dpi) and calculated with the formula S = π×a×b/4, where a and b represent the long and short diameters of the elliptic-like lesion areas, respectively. This assessment experiment was conducted with three replications for each of the 190 DH lines sown in two growing seasons of 2010–2011 and 2011–2012.

The second procedure was for stem inoculation with mycelial agar plugs, which measured stem resistance (SR) at the mature plant stage. Plants in disease nursery plots were inoculated one week after the termination of flowering, which represents the prevalent stage of Sclerotinia stem rot in natural field conditions. Eight to ten stems of each DH line in each replicate were inoculated with mycelial agar plugs at a height of 50 cm above the ground. Each plug was affixed with plastic wrap to ensure close contact of the inoculums with the stem surface and to maintain humidity. The plants were sprayed with water mist every day after inoculation for three days. The lesion length along the stems was measured at 7 dpi.

### Molecular Marker and Linkage Map Construction

Genomic DNA was extracted from young leaves of the parental lines and HJ-DH lines with the procedure as described [22].

Simple sequence repeat (SSR) markers were used in linkage map construction. Parts of the SSR markers used in this study have been previously described [23] and some were developed for this study (Table S1). Primers pairs prefixed by BEN, BGO and BGR were developed from *B. napus* EST sequences, *B. oleracea* and *B. rapa* genome sequences, respectively.

Linkage analysis with all markers was performed using MAPMAKER 3.0. A minimum log likelihood of the odds (LOD) score of 9.0 and a maximum distance of 30 cM were used to classify loci into linkage groups (LGs). The order within each LG was determined by the commands of order, try, and ripple. Assignment of LGs was based on common marker loci from *B. napus* mapping populations as described previously [24]–[28] (http://www.brassica.info/resource/markers/ssr-exchange.php). Genetic distances between loci were calculated using the Kosambi mapping function [29].

### QTL Mapping and Statistical Analysis

QTL analysis was performed by composite interval mapping (CIM) [30] using the Windows version of QTL cartographer 2.5 software (http://statgen.ncsu.edu/qtlcart/WQTLCart.htm). Forward and reverse regression analysis was applied for QTL detection. Cofactors were selected by the program using Model 6 with genetic background controlled by five markers, window size set at 10.0 cM and probability for into and out set at 0.05. Significance thresholds at the 0.05 significance level were estimated on the basis of 1,000 permutations using the procedure as described previously [31]. The confidence interval of QTL was determined by 1-LOD intervals surrounding the QTL peak. QTLs detected in different environments were considered to be the same if they had overlapped confidence intervals.

Epistatic interactions among loci were estimated using QTLNetwork 2 based on a mixed-model approach [32]. The 2D genome scans were conducted with a significance level of p<0.05 based on 1,000 permutations.

The heritability (*h^2^*) of LR and SR was calculated as *h* ^2^ = σ^2^_g_/(σ^2^_g_+ σ^2^_gl_/n+ σ^2^_e_/nr), where σ^2^_g_ is the genotypic variance, σ^2^_gl_ is the variance due to genotype by environment interaction, σ^2^_e_ is the error variance, n is the number of environments, and r is the number of replications. The estimates of σ^2^_g_, σ^2^_gl_ and σ^2^_e_ were obtained from an analysis of variance (ANOVA) using the general linear model (GLM) procedure in the SAS software (SAS Institute 2000) with environment considered as a random effect.

### Comparative Mapping of Linkage Group C6 with Arabidopsis and *B. oleracea*

A procedure described by Cai et al. (2012) was used for comparative mapping between *B. napus* and Arabidopsis with SSR markers through *B. rapa* and *B. oleracea* genome sequences [33]. Electronic PCR (e-PCR) [34] was performed to locate the SSR marker primer sequences of LG C6 on the *B. oleracea* genome (http://www.ocri-genomics.org/bolbase/) and to identify the homologous colinear loci in *B. oleracea*. The e-PCR amplicons’ sequences in *B. oleracea* genome were used as queries in the search by using BLASTn [35] against TAIR10 (http://www.arabidopsis.org/) with an E-value of 1E-10. The positions and gene loci of best-hits in Arabidopsis genome sequences database were collected and comparatively mapped on the *B. napus* LGs. The conserved Arabidopsis genome blocks were divided and named as described previously [36].

### Gene Cloning and Expression Analysis

To identify all putative *IGMT5* copies in *B. napus* genome, we searched for the sequences homologous to Arabidopsis *AtIGMT5* (At1g76790) gene in the databases of *B. rapa* (http://brassicadb.org/brad/index.php) and *B. oleracea* (http://www.ocri-genomics.org/bolbase/). The search was conducted first with the BLASTn program using an E value <1E-20 and identity and query coverage >80%. All hits from this search were then used to locate the homologous sequences in the NCBI nucleotide collections (http://www.ncbi.nlm.nih.gov) with the same parameters as above. The nonredundant sequences resulted from the hits were then collected and compared with known *AtIGMT5* in Arabidopsis.

Primers were designed to amplify the genomic fragments of Arabidopsis *AtIGMT5* homologues in *B. napus* based on sequence information in *B.rapa* and *B.oleracea* genomes. The PCR products were cloned into pMD18-T vector (Takara Corporation, Japan) according to the manufacturer’s instructions. The M13F and M13R universal primers and the BigDye Terminator Cycle Sequencing v3.1 (Applied Biosystems, Foster City, CA, USA) were used for sequencing. Sequences were aligned using the computer program SEQUENCHER 4.1.2 (Gene Codes Corporation, Ann Arbor, MI, USA).

For gene expression analysis, the main stem of plants for disease challenge was inoculated at three sites (every other internode) at 40–60 cm above the ground with mycelial agar plugs, while mock plants were treated with an agar plugs at three sites only. Epidermal stem tissues extending 10 mm beyond the inoculation site and 1 mm deep were excised at 24, 48, 72 and 96 hours post inoculation (hpi) with a razor blade [18]. Three biological replicates were collected and the tissues harvested from three individual plants (9 inoculation sites in total) were pooled as one biological replicate for each time point. The sampled tissues were immediately frozen in liquid nitrogen and stored at −80°C before RNA preparation.

Total RNA was prepared from the sampled tissues with Trizol reagent (Invitrogen, USA). For each sample, 5 µg RNA was digested with 1 µl of DNase (Thermo, USA) to exclude residuary DNA, and was then used for reverse transcription reaction with TransScript First-Strand cDNA Synthesis Super Mix (TransGen, China). Allele-specific primer pairs of MT5 (5′-CTGGATTCAGCGTTGGAGTTA-3′ and 5′-GTGAACTCAAGATCCATGAAACT-3′), were used to analyze the expression of *BnaC.IGMT5.a*.

## Results

### Phenotypic Evaluations

**Figure 1:**
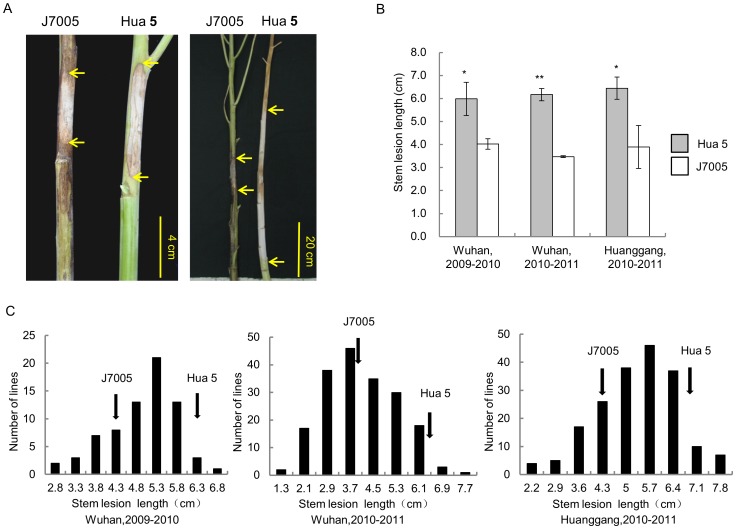
Stem resistance (SR) of the two parental lines, Hua 5 and J7005, and the HJ-DH population. (A) Disease lesion on the stem of Hua5 and J7005 at 7 dpi (left panel) and 30 dpi (right panel). Arrows indicate the lesion boundaries on the stem. (B) Lesion length of Hua5 and J7005 at 7 dpi. * and ** indicates a significant difference at P<0.05 and P<0.01 levels, respectively. (C) Frequency distributions of the lesion length in the HJ-DH population in Wuhan, 2009–2010 (left), Wuhan, 2010–2011(middle) and Huanggang, 2010–2011 (right). Arrows indicate the mean lesion length of the parental lines.

**Figure 2:**
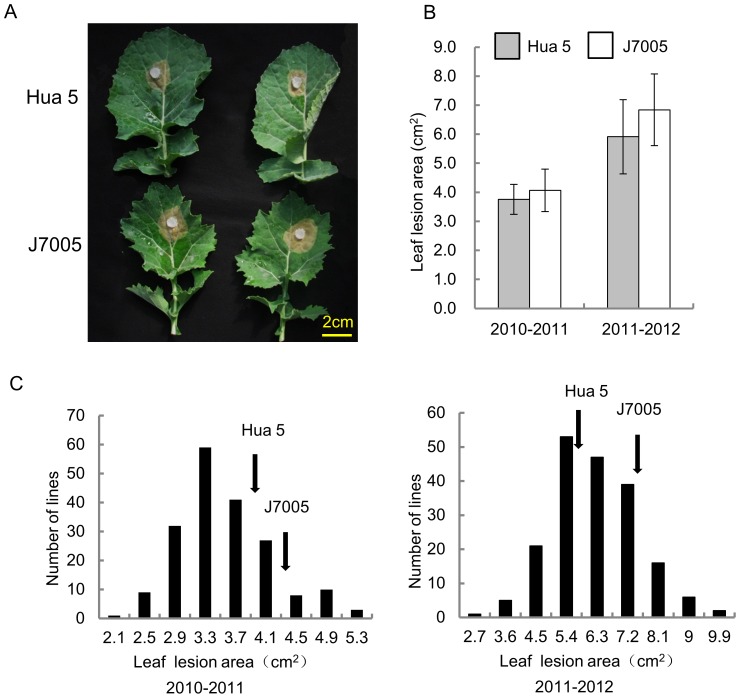
Leaf resistance (LR) of the two parental lines, Hua 5 and J7005, and the HJ-DH population. (A) Disease lesion on the leaf of Hua5 and J7005 at 2 dpi. (B) Lesion area of Hua5 and J7005 at 2 dpi. * and ** indicates a significant difference at P<0.05 and P<0.01 levels, respectively. (C) Frequency distributions of the lesion area in the HJ-DH population in the season of 2010–2011 (left) and in 2011–2012 (right). Arrows indicate the mean lesion size of the parental lines.

The resistance performance of the parental lines and the HJ-DH population was assayed at two developmental stages. For stem resistance (SR) at the mature stage, a significant difference of lesion length on stem at 7 dpi between the two parents was observed (Fig. 1A, B). The lesion extended further to most part of the stem of Hua 5 and led to death at 30 dpi, while the lesion was restrained to about 10–15 cm on the stem of J7005 (Fig. 1A). Transgressive segregation and continuous distribution in the HJ-DH population were observed for SR in three environments (Fig. 1C). For leaf resistance (LR) at the seedling stage, no significant difference between the two parental lines was detected (Fig. 2A, B). However, the HJ-DH population showed a similar transgressive segregation to SR in two years (Fig. 2C). The segregation patterns for both SR and LR in the mapping population implied that resistance to *Sclerotinia* infections was a quantitative trait with additive gene effects.

The correlation coefficients across seasons and environments for both the SR and LR assessments were significant (P<0.01; Table S2), indicating that the inoculation procedures were reliable. A two-way ANOVA for LR and SR at two environments with 190 DH lines (The SR assay data from the season of 2009–2010 with 71 DH lines were not included) showed that the differences among the genotypes and environments for LR or SR were highly significant (Table 1). A significant genotype-by-environment interaction was detected for SR, but not for LR (Table 1). The heritability (*h* ^2^) of the LR and SR was as high as 61.01% and 68.31%, respectively (Table 1), indicating that genetic variance accounted for a large portion of the phenotypic variance of resistance to *S. sclerotiorum*.

**Table 1:** A two-way ANOVA analysis and broad sense heritability (*h^2^*) for stem rot resistance at two developmental stages in the HJ-DH population.

Traits	Variation	*df*	MS	*F*	P	*h* ^2^(%)
LR	Replication	2	156.74	64.55	0.0000	61.01
	Genotype (G)	189	3.84	1.58	0.0000	
	Environment (E)	1	1623.50	668.63	0.0000	
	G×E	189	1.50	0.62	0.9999	
	Error	758	2.43			
SR	Replication	2	151.91	87.50	0.0000	68.31
	Genotype (G)	189	6.84	3.94	0.0000	
	Environment (E)	1	515.35	296.84	0.0000	
	G×E	189	2.17	1.25	0.0230	
	Error	758	1.74			

A significant positive correlation (r = 0.18–0.46, p<0.01) between LR and SR was found in most of the environments (Table S2), suggesting that LR evaluated by detached leaf inoculation could be used as an indicator for SR. Observations on three key growth periods (bolting, budding and flowering time) in 2010–2011 growing season showed that there was no significant correlation between the three key growth periods and SR (Table S3), suggesting that the detected SR in the HJ-DH population was mainly a result of genetic variation among the genotypes rather than disease escape.

### Mapping of QTLs for *S. sclerotiorum* Resistance

A total of 272 molecular markers corresponding to 302 SSR loci were mapped onto 19 LGs in the DH population, covering a genetic distance of 1,579 cM with an average interval of 5.2 cM between adjacent markers according to the Kosambi function [29]. The LGs corresponded to the 19 chromosomes of *B. napus* including A1–A10 (A genome) and C1–C9 (C genome), as determined by shared SSR markers with public genetic maps in the literature (http://www.brassica.info/resource/maps/lg-assignments.php). All SSR markers were evenly distributed across the whole genome of *B. napus* with 147 and 155 SSR loci on A and C genome, respectively.

**Figure 3:**
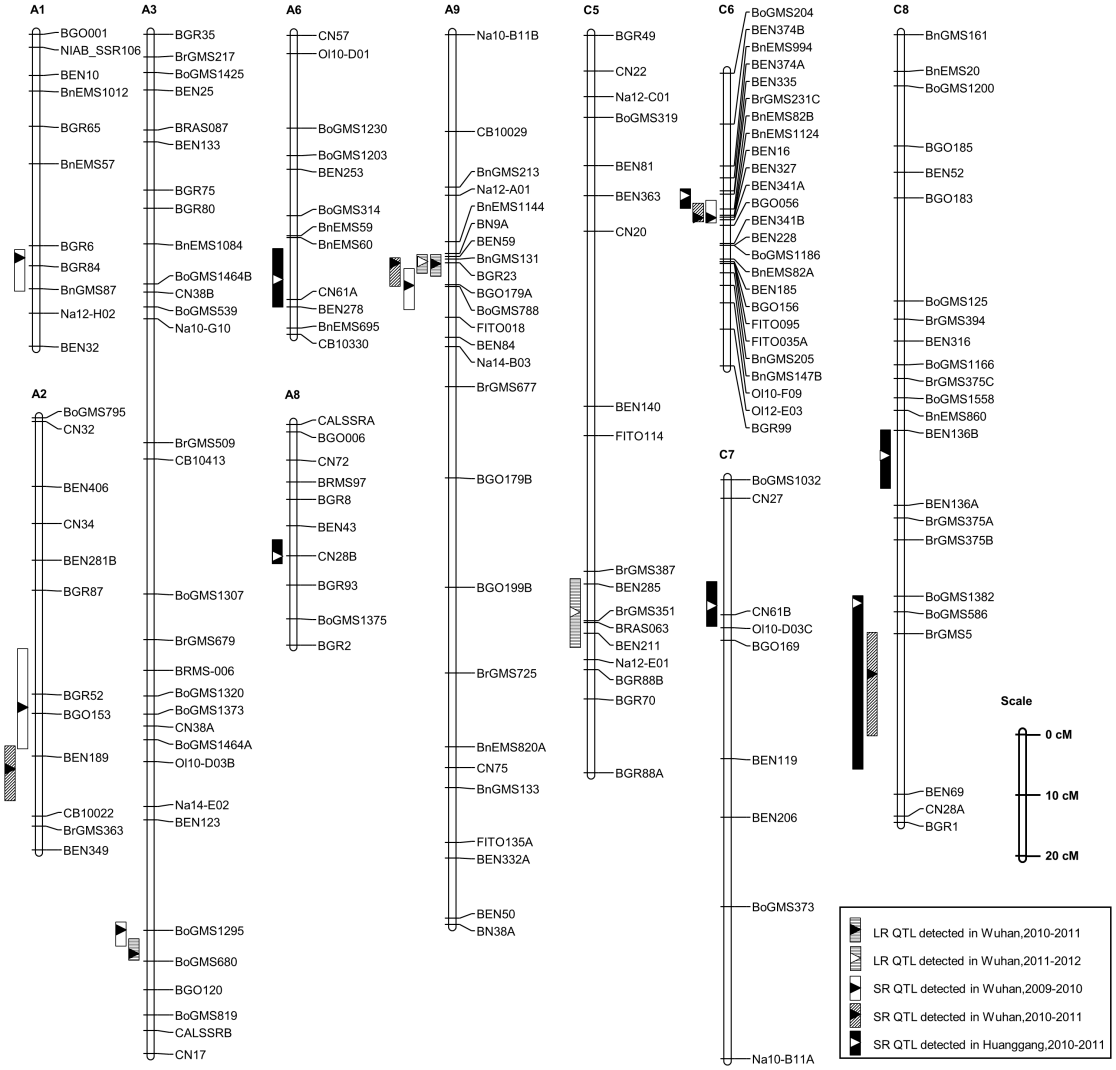
QTLs for LR and SR mapped on the HJ-DH genetic linkage map. The bar to the left of the LG indicates the 1-LOD confidence interval for the QTL and the triangle indicates the QTL peak position.

A total of 13 QTLs for LR and SR were identified (Table 2; Fig. 3). Among the QTLs, 3 for LR were mapped on LG A3, A9 and C5, and 10 for SR mapped on 9 LGs (A1, A2, A3, A6, A8, A9, C6, C7 and C8), respectively. Notably, a major QTL *LRA9* for LR was identified in both growing seasons, which accounted for 15.86% of the trait variation in 2010–2011 and 8.54% in 2011–2012 (Table 2; Fig. 3). The allele from J7005 at this locus increased the leaf resistance to *S. sclerotiorum*. The remaining two QTLs (*LRA3* and *LRC5*) for LR were detected in only one growing season with their resistant alleles from Hua 5 (Table 2).

**Table 2:** Putative QTLs for LR and SR in the HJ-DH population.

Traits	Location(Season)	QTL^a^	Peak^b^	LOD	A^c^	R^2^(%)^d^	CI (cM)^e^
**LR**	Wuhan(2010–2011)	*LRA3*	151.1	4.96	−0.20	7.91	148.7–151.9
		*LRA9*	37.6	9.29	0.28	15.86	36.2–39.7
	Wuhan(2011–2012)	*LRA9*	37.1	3.81	0.40	8.54	36.2–38.2
		*LRC5*	94.5	3.29	−0.37	7.28	89.1–100.8
**SR**	Wuhan(2009–2010)	*SRA1*	36.7	3.90	0.48	14.33	36–42.1
		*SRA2*	47.6	3.45	0.34	14.11	36.9–54.3
		*SRA3*	147.4	5.77	−0.46	19.03	147.2–149.5
		*SRA9*	41.3	2.96	0.23	6.82	38.3–45.2
		*SRC6*	23.8	10.06	−0.56	29.01	20.9–24.6
	Wuhan(2010–2011)	*SRA2*	57.9	4.57	0.43	10.36	54.2–62.8
		*SRA9*	37.6	6.25	0.48	13.07	37.1–41.5
		*SRC6*	23.8	19.38	−0.74	30.14	21.5–24.3
		*SRC8a*	108.5	4.38	0.44	9.95	98.3–114.3
	Huanggang(2010–2011)	*SRA6*	41.3	3.83	0.34	5.44	36.3–44.5
		*SRA8*	21.8	6.95	0.41	8.23	19.4–22.2
		*SRC6*	20.0	22.93	−0.72	32.61	19–22.3
		*SRC7*	21.2	4.54	−0.36	12.16	14.9–24.4
		SRC8b	69.1	4.66	0.40	7.02	65.2–73.5
		*SRC8a*	93.8	3.28	0.27	3.43	92.4–120.7

a QTL nomenclature uses the trait name followed by the LG number; an alphabetical letter a or b or c is added if more than one QTL are identified in one LG.

b *Peak* map position (cM) of peak LOD scores.

c *A* additive effect: positive additivity indicates that the QTL allele originated from the parental line J7005 increase resistance; negative additivity means that the QTL alleles originated from the parental line Hua 5 increase resistance.

d *R^2^* proportion for the phenotypic variation explained by the QTL.

e *CI* Confidence intervals were obtained by marking positions ±1 LOD from the peak.

A major QTL, *SRC6*, which explained 29.01%, 30.14%, 32.61% of the phenotypic variation for SR in the three environments, was detected in LG C6 (Table 2; Fig. 3). Moreover, three QTLs (*SRA2*, *SRA9* and *SRC8a*) were detected in two of the three environments, while the remaining six QTLs for SR were detected in only one environment (Table 2; Fig. 3). Seven of the 10 SR QTLs had their resistant alleles from J7005 (the resistant parent), while three others, including *SRC6*, from Hua 5.

Two QTLs on LG A3, *LRA3* and *SRA3*, both of which had their resistant alleles from Hua 5, had an overlapping confidence interval, while *LRA9* and *SRA9* on LG A9 with resistant alleles from J7005 were also located in the same confidence interval (Table 2; Fig. 3). The association of these two regions on LG A3 and LG A9 with resistance at different developmental stages (LR and SR) suggested that common loci or genes might be involved in the resistance at different developmental stages. The mapping results were consistent with the observation that there was a significant positive correlation between LR and SR (Table S2).

No significant epistatic interaction was detected in SR, while an epistatic interaction that showed significant additive-by-additive effect was found in LR. However, the epistatics only explained 2.14% of the phenotypic variation and was between a pair of non-QTL loci, implying that the QTLs for resistance to *S. sclerotiorum* detected in this HJ-DH population may primarily include genes with additive effects.

### The Effects of *SRC6* and *LRA9* on S*clerotinia* Resistance

**Figure 4:**
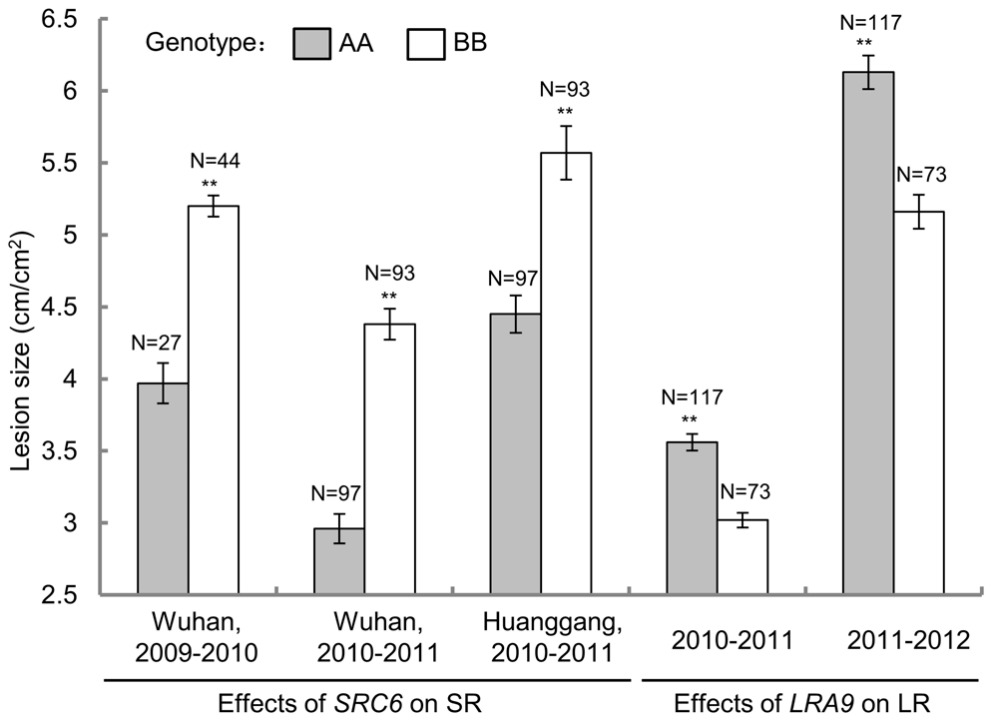
Effects of the major QTL *SRC6* and *LRA9* on SR and LR. Each group (AA and BB) for the DH population is classified according to the genotype of the makers (BEN327 for *SRC6*, BGR23 for *LRA9*) that closely linked to the peaks of the major QTLs. * and ** indicate a significant difference between AA and BB group at P<0.05 and P<0.01 levels, respectively.

To examine the effects of the major QTLs *SRC6* and *LRA9* on disease resistance, the HJ-DH population was classified into two groups according to the genotypes of closely linked markers (BEN16 and BGR23, Fig. 3). For *SRC6*, the average lesion length in the group with the allele from Hua 5 (genotype AA) was significantly shorter than that of J7005 (genotype BB) in all three environments, with the lesion length reduced by 20.1–32.4% at 7 dpi (Fig. 4). For *LRA9*, the lesion area of the group containing the allele from J7005 (BB) was significantly smaller than that of AA group, with the lesion area reduced by 15.2–15.8% at 2 dpi (Fig. 4).

### Comparative Mapping of C6 Linkage Group with Arabidopsis and *B. oleracea*

**Figure 5:**
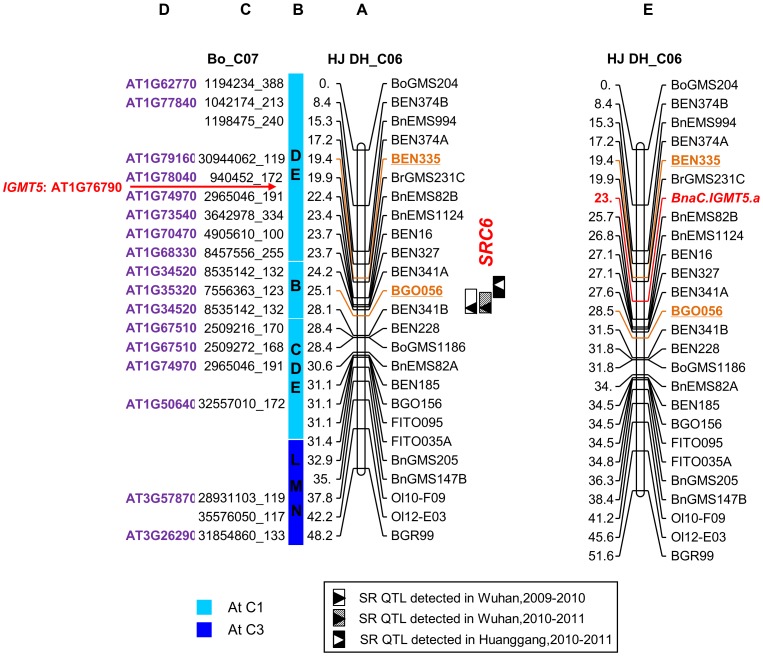
Comparative map of LG C6 of *B.* *napus* with *B. oleracea* and Arabidopsis. Column A presents the linkage map of LG C6 of the HJ-DH population. The confidence interval of *SRC6* is shown in color. Column B is the conserved blocks identified in *B. napus*, which is labeled according to Schranz et al. [36] and colored differently based on the Arabidopsis (At) chromosome positions defined by Parkin et al. [56]. Column C lists the homologous colinear loci in *B. oleracea* chromosome 7 (BoC7) corresponding to SSR markers in LG C6. The number designates the physical position in *B. oleracea* chromosome with the size of amplification fragments. Column D lists the genes encoding homologous loci in *A. thaliana*. Column E presents the modified LG C6 after adding *BnaC.IGMT5.a* on the map.

To predict the candidate genes for *SRC6*, a comparative map of LG C6 with Arabidopsis was constructed based on *B. oleracea* genome sequences using a previously described procedure [33]. With the SSR markers primers on LG C6, electronic PCR (e-PCR) was performed with the genome sequence of *B. oleracea* used as templates to obtain fragments amplified in *B. oleracea* genomes (amplicons). In total, 19 of the SSR loci were aligned to chromosome 7 (BoC7) (Fig. 5). To validate the results of e-PCR, two amplified fragments with SSR markers (BrGMS231C and BEN327) in the confidence interval of *SRC6* were cloned and sequenced. The cloned fragment was aligned to Scaffold000076 and Scaffold000096 on BoC7 of the *B. oleracea* genome. Comparative analysis revealed that LG C6 was co-linear with BoC7 (Fig. 5), which was consistent with the findings in previous studies [33], [37]. By BLASTn analysis against TAIR10 with these e-PCR amplicons’ sequences, 15 and 2 amplicons were matched to homologous loci from Arabidopsis chromosome 1 and 3 (AtC1 and AtC3), respectively (Fig. 5). Three Arabidopsis conserved blocks (D, E and B) were identified to correspond with the confidence interval of *SRC6* (19–24.6 cM), and the peak of the *SRC6* fell in block E (Fig. 5).

### Screening of Candidate Genes for *SRC6* through Bioinformatic Analysis

In order to further narrow down the candidate genes for *SRC6* among the genes located in the mapped region mentioned above, we conducted bioinformatics analysis on genes with significant changes in response to *S. sclerotiorum* challenge in *Brassica* species from public gene profiling databases. In a microarray study, Zhao et al. (2009) investigated gene expression patterns after stem inoculation with *S. sclerotiorum* in two *B. napus* cultivars with different *Sclerotinia* resistance, ZhongYou 821 and Westar [18]. Among the genes with significant expression changes in ZhongYou821 (the resistant cultivar), we noticed that *IGMT5*, a putative Arabidopsis homologue of At1g76790 in *B. napus*, was located in the confidence interval of *SRC6* (block E). Expression of the *IGMT5* gene was induced within 6 hour post-inoculation (hpi), and its expression level increased up to 31.1-fold at 72 hpi (Fig. S1), while the same gene in Westar (the susceptible one) did not show any significant change after inoculation [18]. This difference in expression patterns suggested that the gene may be involved in response to *S. sclerotiorum* infections. Gene annotation based on Arabidopsis genome information showed that *IGMTs* encodes indole glucosinolate methyltransferase (IGMT), which was similar to caffeic acid O-methyltransferase (COMT) [38].

### Identification of the *IGMT5* Gene in *Brassica* Species

To have a complete understanding of the *IGMT5* gene in *B. napus* and its two progenitor species, we searched for all possible copies of *IGMT5* in A-genome (*B. rapa*) and C-genome (*B. oleracea*) in databases using Arabidopsis *IGMT5* gene sequence (At1g76790) as a query. Two copies of the *IGMT5* gene were identified in *B. rapa* and *B. oleracea*, respectively. The copies of the gene were named *BraA.IGMT5.a*, *BraA.IGMT5.b*, *BolC.IGMT5.a* and *BolC.IGMT5.b*, respectively (Table 3). Both *BraA.IGMT5.a* and *BraA.IGMT5.b* were located on A7 with a distance of 6.6 Mb in *B. rapa*, while both *BolC.IGMT5.a* and *BolC.IGMT5.b* were located on C7 with a distance of 30.2 Mb in *B. oleracea* (Fig. S2).

**Table 3:** Genome distributions of *IGMT5* in *B. napus*, *B. rapa* and *B. oleracea*.

Gene identity^a^	Chr^b^	Accession	Source	Forward primer sequence(5′to3′)	Reverse primer sequence(5′to3′)
*BnaA.IGMT5.a*	A7^c^	KC768107	This study	ATTAGCTTCACCGAGGAGAAAGC	CATTACCATGAGACATTACAGCCT
*BraA.IGMT5.a*	A7	Bra015719	BRAD		
*BnaC.IGMT5.b*	C6 c	KC768109	This study	ATACTTAGCAATTCGACGCCTAC	GTGAACCCAAGAACCATACAACT
*BolC.IGMT5.b*	C7	Bol039840	Bolbase		
*BnaA.IGMT5.b*	A7 c	KC768110	This study	GCTATAACATTAGCAACTAGGTACG	CTATTTAGAGAACTCAATGACCCAC
*BraA.IGMT5.b*	A7	Bra003707	BRAD		
*BnaC.IGMT5.a*	C6 c	KC768108	This study	GGAACCACAAGATGCATCTAGAG	AAATGTGAACTCAAGATCCATG
*BolC.IGMT5.a*	C7	Bol027603	Bolbase		

a Gene nomenclature follows the rules proposed by Østergaard and King [57].

b *Chr* chromosome. The chromosome locations of the *IGMT5* genes from *B. rapa* and *B. oleracea* are based on the sequencing information at BRAD (http://brassicadb.org/brad/index.php) and Bolbase (http://www.ocri-genomics.org/bolbase/).

c The Chromosome location from *B. napus* is deduced based on the synteny of *B. napus* and its two progenitor species, *B. rapa* and *B. oleracea*.

**Figure 6:**
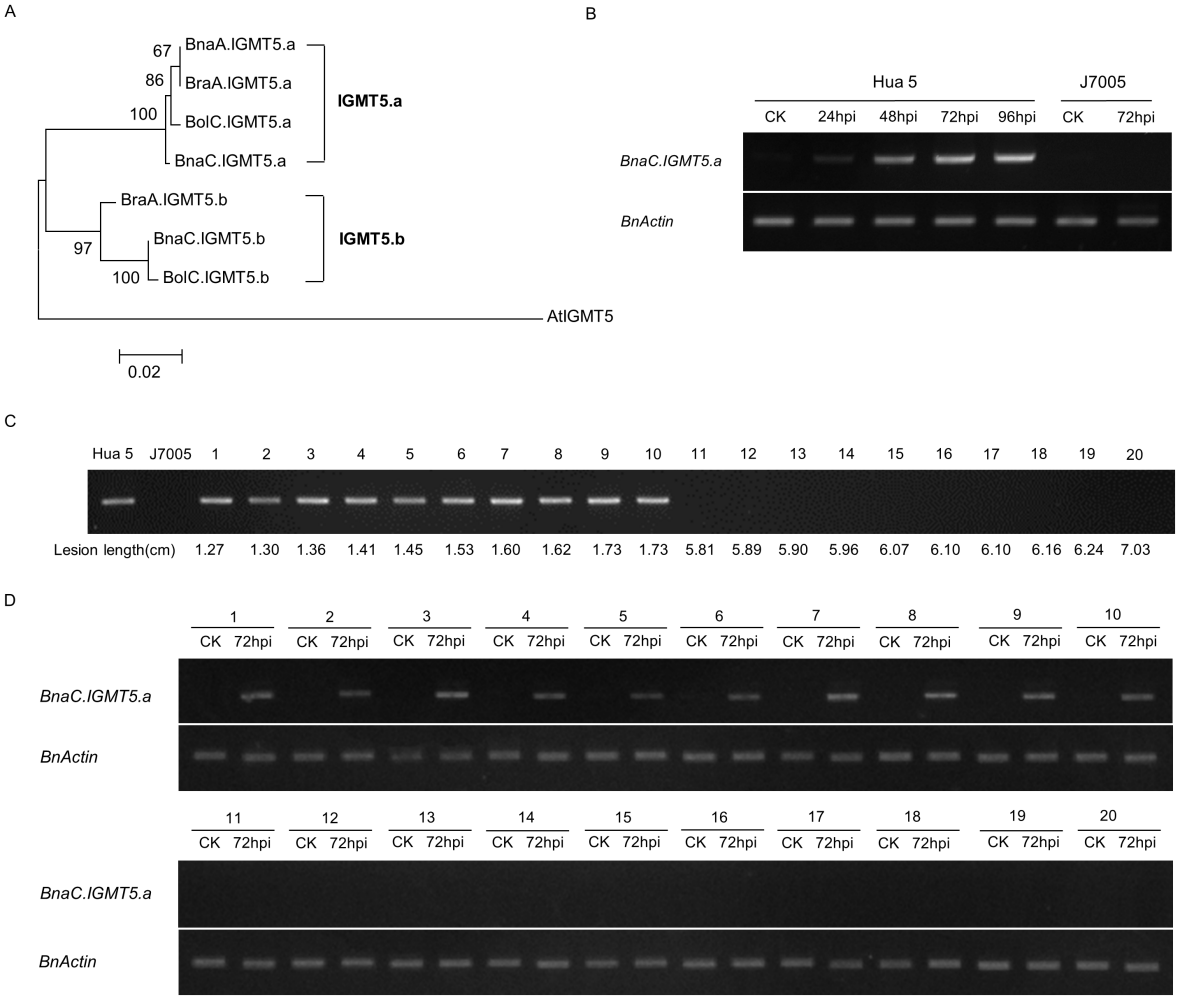
Molecular cloning of *IGMT5* genes in three *Brassica* species and induced expression of *BnaC.IGMT5.a* after inoculation with *Scelrotinia* pathogen. (A) Phylogenetic analysis of *IGMT5* genes in *B. rapa*, *B. oleracea* and *B. napus*. Neighbor joining tree is presented based on the deduced amino acid sequences of *IGMT5* genes in three *Brassica* species. Bootstrap values (1,000 replications) are shown at each branch as a percentage. A branch length scale bar is shown beneath each tree. (B) *BnaC.IGMT5.a* expression is induced in Hua 5 after inoculation with *Sclerotinia* pathogen, but not induced in J7005. RT-PCR analysis was conducted with RNAs from pooled tissues after inoculations at each time point. *BnActin* was used as an internal control. (C) *BnaC.IGMT5.a* is associated with resistant phenotype. PCR products amplified from the copy-specific marker of *BnaC.IGMT5.a* are presented. Lane 1–10 are the samples from most resistant lines and lane 11–20 the most susceptible lines from the HJ-DH population. (D) RT-PCR analysis of *BnaC.IGMT5.a* in the ten most resistant lines (lane 1–10) and ten most susceptible lines (lane 11–20) at 72 hpi.

Based on the sequence information of the *IGMT5* genes in *B. rapa* and *B. oleracea*, putative genomic fragments of *IGMT5* in *B. napus* were isolated through a homologous cloning strategy from Hua 5 and J7005. Four genomic DNA sequences of *IGMT5* were identified after PCR amplification, molecular cloning and sequencing. The cloned four *IGMT5* nucleotides in *B. napus* were then named *BnaA.IGMT5.a*, *BnaA.IGMT5.b*, *BnaC.IGMT5.a* and *BnaC.IGMT5.b*, respectively. The four putative genes were located on A7, A7, C6 and C6 chromosome of *B. napus*, respectively, based on the synteny of *B. napus* and its two progenitor species, *B. rapa* and *B. oleracea* (Table 3; Fig. 6A; Fig. S3). Among the four copies of *IGMT5* in *B. napus*, *BnaA.IGMT5.b* had a 2773 bp deletion at the first exon and the 5′ untranslated regions (UTR) compared with *BraA.IGMT5.b* (Fig. S4). As such, no integrated cDNA of *BnaA.IGMT5.b* could be amplified, suggesting that it is a pseudogene in *B. napus*.

Phylogenetic analysis was performed based on the predicted protein sequences of *IGMT5* genes identified in this study to reveal the evolutionary relationship among the copies. The three intact *IGMT5* members from *B. napus* and their corresponding counterparts in two diploid species could be grouped into two clusters (*IGMT5.a* and *IGMT5.b*; Fig. 6A). Members belonging to each of the two categories were more distantly related than the members within a same category (Fig. 6A). For example, *BnaA.IGMT5.a* and *BraA.IGMT5.a* were more closely related to each other than members from the same species such as *BraA.IGMT5.a* and *BraA.IGMT5.b.*

### *BnaC.IGMT5.a*, is Involved in the Defense Reaction against *S. sclerotiorum* Infection and a Candidate Gene for *SRC6*

To establish the relation of the identified *IGMT5* gene and *SRC6*, we compared the sequence differences of all the copies of the gene between the two parental lines. For both of *BnaA.IGMT5.a* and *BnaC.IGMT5.b*, there was no sequence difference between Hua 5 and J7005, while the pseudogene, *BnaA.IGMT5.b* on A7, had 5 single nucleotide polymorphisms (SNPs) between Hua 5 and J7005 (Fig. S3). Due to its psudogene nature, such a difference was not likely to cause a phenotypic variation.

Another copy in LG C6 (*BnaC.IGMT5.a*) could be amplified as an integrated gene structure in Hua 5, while not be amplified in J7005 even with different primers, suggesting that *BnaC.IGMT5.a* in J7005 may have been deleted or inserted with a large fragment. To further test such an inference we developed an allele-specific marker using primer pairs of MT5 (5′-CTGGATTCAGCGTTGGAGTTA-3′ and 5′-GTGAACTCAAGATCCATGAAACT-3′), which were developed based on the genomic sequence and putative CDS of *BnaC.IGMT5.a*. Using the HJ-DH population, the gene was mapped between the marker BrGMs231C (corresponding to At1g78040) and BnEMS82B (corresponding to At1g74970) (Fig. 5), the confidence interval of *SRC6*, which was consistent with the comparative mapping results (Fig. 5).

We then investigated the expression of *BnaC.IGMT5.a* after inoculation with *S. sclerotiorum* by RT-PCR analysis with allele-specific primer pairs MT5. The expression of *BnaC.IGMT5.a* in Hua 5, the donor line of the resistant allele was very low at normal growth conditions (without pathogen inoculation). However, the expression was induced dramatically at 24 hpi compared with mock-inoculated control, and the expression levels continued to increase from 24 hpi to 96 hpi (Fig. 6B). This result was consistent with the microarray data from Zhongyou 821 (Fig. S1). However, we did not detect any expression of the gene in J7005, the parental line lacking the allele under the same condition (Fig. 6B).

The specificity of the allele-specific primer pairs MT5 was further validated with ten most resistant lines and ten most susceptible lines from the HJ-DH population. All the resistant lines had a band representing the *BnaC.IGMT5.a* gene, while the susceptible lines lacked this copy (Fig. 6C), indicating that the allele contributed to resistance significantly. Furthermore, we analyzed the gene expression of *BnaC.IGMT5.a* in ten most resistant lines after inoculation with the pathogen. Compared with mock-inoculated control, all the ten most resistant lines exhibited a significant increase of *BnaC.IGMT5.a* mRNA (Fig. 6D). However, no such an inducible increase of mRNAs could be detected in ten susceptible lines under the same analytic conditions for gene expression (Fig. 6D).

Taken together, the above data suggest that *BnaC.IGMT5.a* is involved in the defense against *S. sclerotiorum* infection in oilseed rape, and likely the candidate gene for *SRC6.*

## Discussion

Our study indicated that the resistance to *S. sclerotiorum* in *B. napus* is a complex quantitative trait and is controlled by minor polygenes, which is consistent with previous reports [7], [8], [11]–[15]. Several other crop species also exhibit such quantitative characteristics in defense against *S. sclerotiorum*, such as soybean [39], common bean [40]–[42], and sunflower [43]–[46]. Thus, it is important to identify *Sclerotinia*-resistance related genes through QTL mapping from the current breeding resources, as the identification of these QTLs and the understanding of the functions and action patterns of the genes for these QTLs provide not only direct gene resources for genetic improvement of *Sclerotinia* resistance, but also the knowledge required for developing effective strategies for *Sclerotinia* resistance breeding.

In the present study, we identified a major QTL, *SRC6*, which explained 29.01%–32.61% of the phenotypic variation in all three environments (Table 2; Fig. 3). To the best of our knowledge, this is the QTL with the largest genetic effect for resistance to *S. sclerotiorum* in *B. napus* reported to date. In a previous study, two QTLs on C6, *Sll16* and *DW16* with overlapping confidence intervals were identified [8]. *Sll16* was detected in three repeated experiments and explained 5.9%–14.9% of the phenotypic variation, while *DW16*, which was detected in only one experiment, explained 12.8% of the phenotypic variation [8]. It is not clear whether the QTLs on C6 identified in the previous and present studies are the same, as there lack shared markers in these genetic maps. The fact that QTLs for the resistance on C6 can be repeatedly mapped suggests the importance of genetic components from *B. oleracea* (C-genome) for *S. sclerotiorum*. This is also consistent with recent studies which found several QTLs in C-genome [47], [48]. Further fine mapping and eventually cloning of *SRC6* identified in this research will offer novel information for understanding the resistance mechanism and provide valuable resources for *Sclerotinia*-resistant breeding, since immune or highly resistant germplasms are not available in *B. napus* and its close relatives.

Currently, progress in QTL cloning in polyploidy crops is still behind that in model plant Arabidopsis and rice due to their complex genome structures. To date, there is no report on a QTL being cloned with map-based cloning procedure in amphidiploid *B. napus*. The issues pertaining to the quantitative resistance such as the resistance to *S. sclerotiorum* in *B. napus* are even more complex because one has to deal with large segregation population and the complexity of plants-microbe-environment interactions to accurately identify the resistance phenotype in a study for QTL fine mapping. Recently, with the rapid advance in sequencing technology, progress has been made in using primary mapping populations to pinpoint the candidate genes for QTLs. For example, ultra-high-density linkage map was used to improve the power and efficiency of genetic analyses and gene discovery in rice [49]–[51]. We have developed a procedure to identify candidate genes of QTLs using a SSR-based *B. napus* genetic map through comparative mapping among Arabidopsis and *B. napus* and its two progenitor species *B. rapa* and *B. oleracea* [33], which circumvents the difficulty of the lack of complete genome sequences in *B. napus.* In the present study, we anchored a candidate gene *BnaC.IGMT5.a* for *SRC6* by means of this procedure together with data mining of microarray expression with pathogen infection.

Several lines of evidence from our study strongly suggest that *BnaC.IGMT5.a* may be the candidate gene for *SRC6*. First, we confirmed through homologous gene cloning that *BnaC.IGMT5a* was the only polymorphic copy among three integrated *IGMT5* copies between parents. In the analysis of the mapping population with allele-specific marker developed based on polymorphism, *BnaC.IGMT5a* was located in the confidence interval of *SRC6* (Fig. 5), thus providing genetic evidence for *BnaC.IGMT5a* as the candidate gene of *SRC6*. Our data showed that *BnaC. IGMT5.a* could be detected as an integrated mRNA coding fragment in Hua 5, the parent with resistance allele, while it could not be amplified in J7005 (Fig. 6C), indicating that the allele may have been deleted or inserted with a great fragment in J7005. Second, expression level of *BnaC.IGMT5.a* in Hua 5 increased significantly at 24 hpi compared with mock-inoculated control, and continued to increase from 24 to 96 hpi by RT-PCR analysis at a 24-hour interval (Fig. 6B). However, there was no detected expression in another parental line of J7500. This gene expression pattern is consistent with the observations in the microarray analysis with resistant cultivar ZhongYou 821 and susceptible Westar [18], in which *IGMT5* expression in ZhongYou 821 was enhanced significantly after being inoculated with *S. sclerotiorum* (Fig. S1) while Westar showed no significant reaction to the inoculation. Third, the availability of *BnaC.IGMT5.a* allele from Hua 5 was associated with disease performance in lines with most resistant or susceptible phenotype from the HJ-DH population (Fig. 6C). Finally, mRNA analysis showed that the expression of *BnaC.IGMT5.a* was obviously induced after inoculation in ten most resistant lines from the population, while the expression was not induced in ten most susceptible lines (Fig. 6D). Taken together, our data and previous studies showed that *BnaC.IGMT5.a* is involved in the molecular mechanism of oilseed rape defense to *S. sclerotiorum*, and is likely the candidate gene for *SRC6.* Given the fact that a large number of disease-resistant QTLs and resistance-related gene expression data are available but have not been fully explored, our strategy in identifying candidate genes for disease-resistant QTL may have wide applications in crop species. It is interesting to note that *IGMT5* is a *COMT*-like gene, which belongs to the gene family involved in the monolignol biosynthetic pathway [38], [52]. Monolignol biosynthesis has been shown to be associated with the resistance to *S. sclerotiorum* in *Camelina sativa* [53] and the resistance to the wilt fungus *Verticillium dahlia* in cotton (*Gossypium spp.*) [54]. Thus, the molecular mechanism of *BnaC.IGMT5.a* contributing to rapeseed defense against *S. sclerotiorum* merits further investigation. Further study is needed to verify the molecular functions of *BnaC.IGMT5.a* in stem rot resistance through more comprehensive investigations including a genetic transformation to validate its function in a susceptible genotype.

In this study, *LRA9*, a major QTL for LR, was identified across years and explained 8.54–15.86% of the total phenotypic variation (Table 2). There was no QTL identified in A9 for *S. sclerotiorum* resistance in *B. napus* in previous studies [7], [8], [15]. In the same region, we also identified a SR QTL, *SRA9*. Furthermore, a QTL region on LG A3 was also found to include both LR and SR. The availability of the two QTL regions associated with both LR and SR may indicate that there are some common loci or genes involved in both LR and SR, and thus partly explain the positive correlation between LR and SR (Table S2). The stem rot occurring at mature plant stage is the major cause of yield loss after infection of *S. sclerotiorum* in oilseed rape. For that reason, our focus on *S. sclerotiorum* resistance is to identify SR QTLs mainly using stem inoculation for assessing the resistance, which is time-consuming and labor-costing. Considering the significant positive correlation between LR and SR, the use of detached leaf inoculation at seedling stage for initial screening and stem inoculation method at mature stage for verification can greatly reduce the workload of inoculation at mature stage and will be beneficial for large scale screening of germplasms in disease breeding.

In this study, we used the disease nursery inoculation for phenotypic evaluation on a large mapping population for stem rot resistance in oilseed rape. Such a procedure allows us to identify the resistant differences among the lines under normal physiological conditions, which is important for the identification of putative resistant QTLs useful for practical breeding. It has been observed that plant architecture and mature date of oilseed may result in disease escape rather than physiological resistance in field screening experiments [55]. Mei et al. (2012) showed significant negative corrections(r = −0.26 to −0.39) between flowering time and *Sclerotinia* resistance in *B. oleracea* [47]. In the present study, we were able to determine *S. sclerotiorum* resistance in the DH population had a small correlation with the growth period (Table S3) in the field-growing condition. Therefore, we conclude that resistance segregation in the HJ-DH population was mainly caused by physiological resistance conferred partly by the genetic components identified in this study.

## Supporting Information

Figure S1 Expression change of *IGMT5* in ZhongYou 821 after *S. sclerotiorum* infection based on the microarray data in Zhao et al. [18].
(TIF)

Figure S2 The distribution of all copies of *IGMT5* in A-genome (*B. rapa*) and C-genome (*B. oleracea*).
(TIF)

Figure S3 Alignment of the genomic nucleotide sequences of all copies of *IGMT5* between Hua 5 (P1) and J7005 (P2).
(DOCX)

Figure S4 Alignment of the genomic nucleotide sequences of *BraA.IGMT5.b* and *BnaA.IGMT5.b*, and the deletion in *BnaA.IGMT5.b*.
(DOCX)

Table S1 Primer sequences of the newly developed SSR markers in the study.
(DOCX)

Table S2 Correlation coefficients of SR and LR in different environments.
(DOCX)

Table S3 Correlation coefficients of SR and three key growth periods (bolting, budding and flowering time) in Wuhan, 2010–2011.
(DOCX)
