# Supplementary figures and images for: Identification of QTLs for Resistance to Sclerotinia Stem Rot and BnaC.IGMT5.a as a Candidate Gene of the Major Resistant QTL SRC6 in Brassica napus
Source: PLoS One. 2013 Jul 2;8(7):e67740. doi: 10.1371/journal.pone.0067740 (PMC3699613; doi:10.1371/journal.pone.0067740)

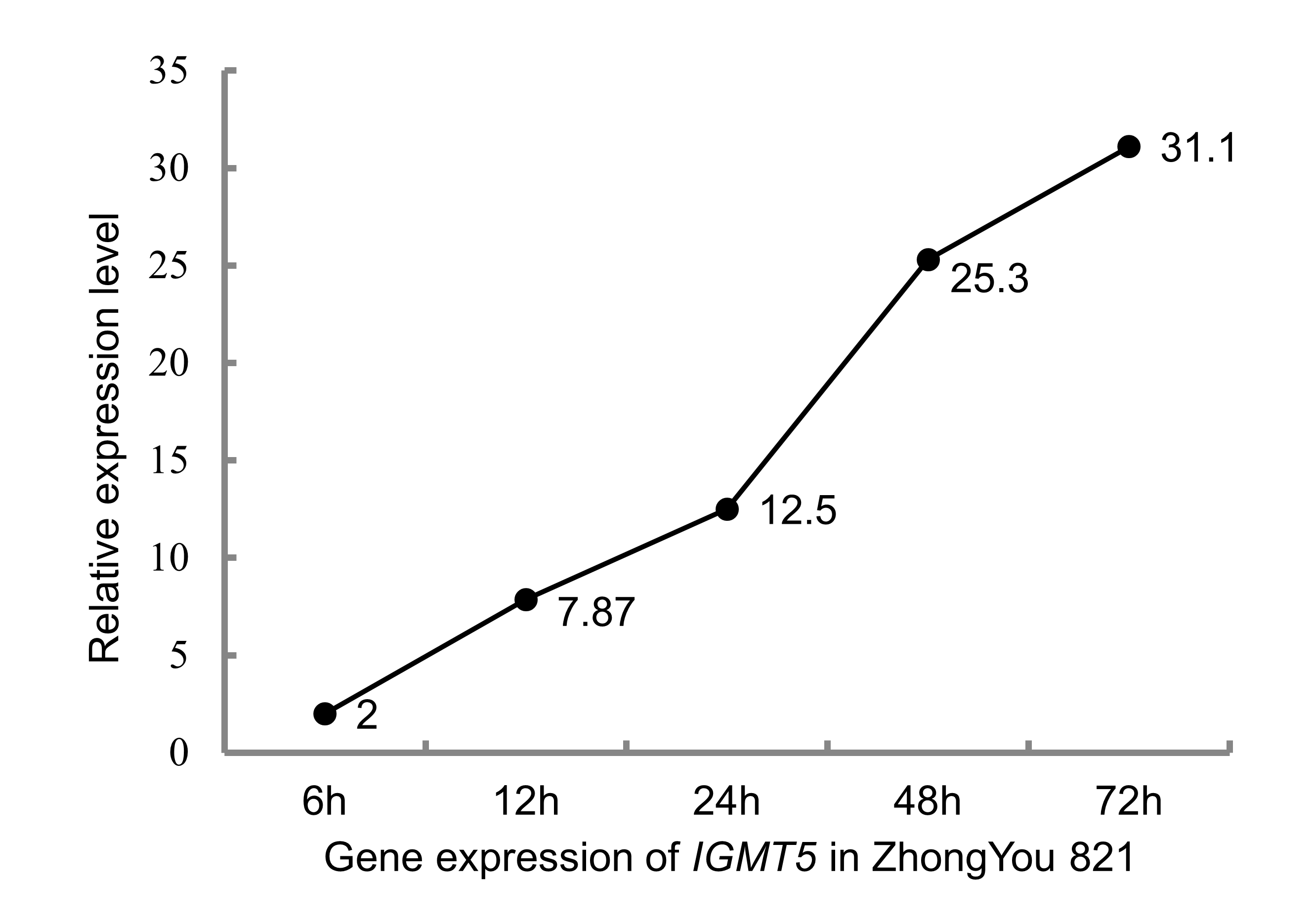

Supplement: Figure S1 — Expression change of IGMT5 in ZhongYou 821 after S. sclerotiorum infection based on the microarray data in Zhao et al. [18]. (TIF) [file pone.0067740.s001.tif]

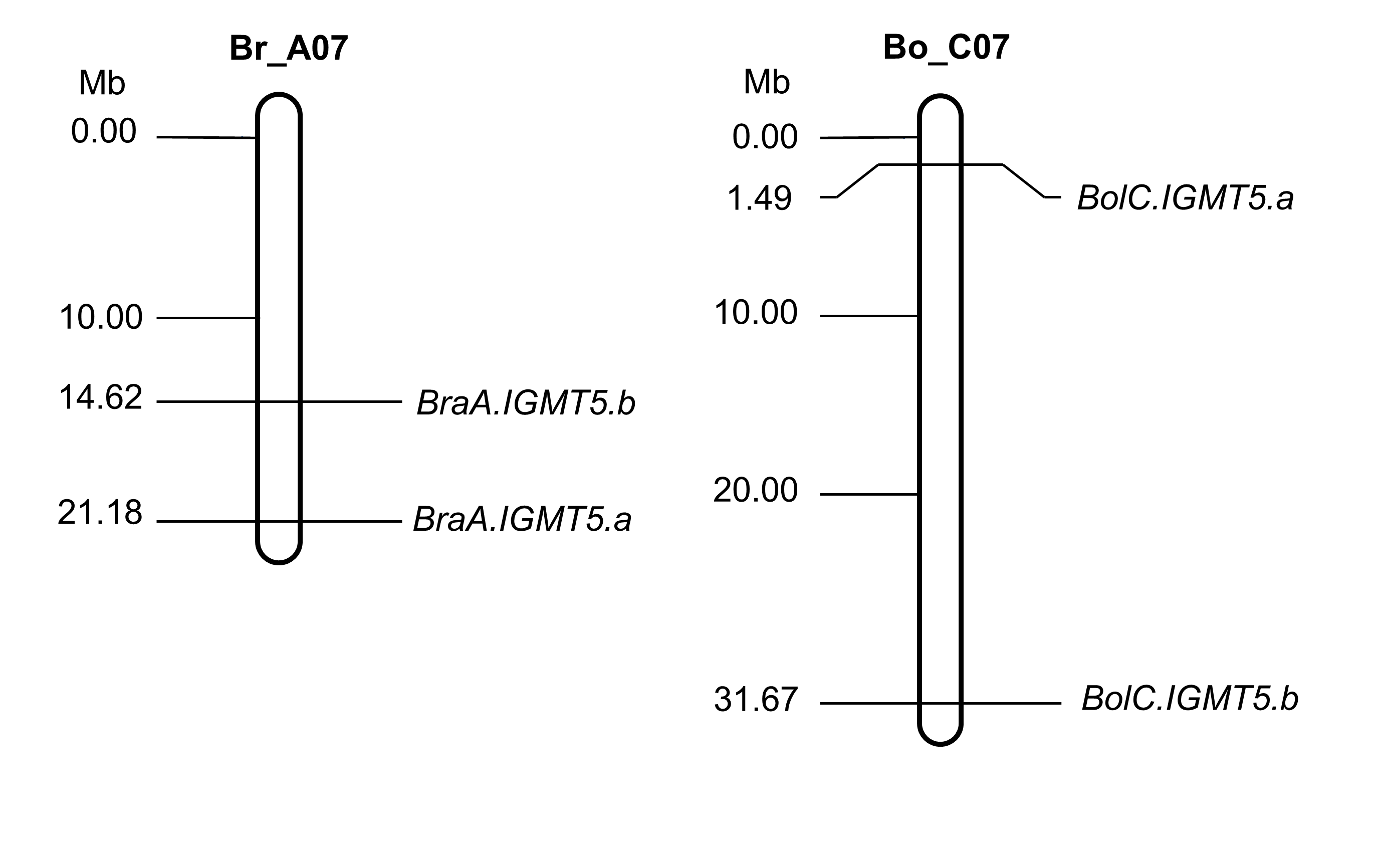

Supplement: Figure S2 — The distribution of all copies of IGMT5 in A-genome (B. rapa) and C-genome (B. oleracea). (TIF) [file pone.0067740.s002.tif]
