# Supplementary material for: Identification of QTLs for Resistance to Sclerotinia Stem Rot and BnaC.IGMT5.a as a Candidate Gene of the Major Resistant QTL SRC6 in Brassica napus
Source: PLoS One. 2013 Jul 2;8(7):e67740. doi: 10.1371/journal.pone.0067740 (PMC3699613; doi:10.1371/journal.pone.0067740)
Supplement: Figure S3 — Alignment of the genomic nucleotide sequences of all copies of IGMT5 between Hua 5 (P1) and J7005 (P2). (DOCX) [file pone.0067740.s003.docx]

***BnaA.IGMT5.a***

BnaA.IGMT5.a-P1 ATGGGACACCTTTTAGACCCTAAAACCATGAATGAGATTAATGGAGATGATGAGACCGAGCTTGGTTTGAGGGCAGTGAGGCTAGCCAATTACATTACCT 100

BnaA.IGMT5.a-P2 ATGGGACACCTTTTAGACCCTAAAACCATGAATGAGATTAATGGAGATGATGAGACCGAGCTTGGTTTGAGGGCAGTGAGGCTAGCCAATTACATTACCT 100

Clustal Consensus **************************************************************************************************** 100

BnaA.IGMT5.a-P1 TCCCCATGGTTTTCAAAGCCGCCATTGAGCTCGGTGTCATCGACGCTCTCTACTTAGCTGCTCGTGATGACGTCAATGGATCTGGATCGTTCCTCAAACC 200

BnaA.IGMT5.a-P2 TCCCCATGGTTTTCAAAGCCGCCATTGAGCTCGGTGTCATCGACGCTCTCTACTTAGCTGCTCGTGATGACGTCAATGGATCTGGATCGTTCCTCAAACC 200

Clustal Consensus **************************************************************************************************** 200

BnaA.IGMT5.a-P1 GTCTGAGATAGCTACTCGGCTTCCCACACCGCCTGGTAACCCTGAAGCACCGGTTTTGCTGGACCGTATGCTTCGTTTACTCGCCAGTTACTCAATGGTC 300

BnaA.IGMT5.a-P2 GTCTGAGATAGCTACTCGGCTTCCCACACCGCCTGGTAACCCTGAAGCACCGGTTTTGCTGGACCGTATGCTTCGTTTACTCGCCAGTTACTCAATGGTC 300

Clustal Consensus **************************************************************************************************** 299

BnaA.IGMT5.a-P1 AAGTGCCAGATAGTAGACGGTGAGAGGGTGTACAAAGCTGAGCCTATTTGTAAGTATTTCTTGAGATACAATATTGAAGAAATGGGGACACTTGCTTCTC 400

BnaA.IGMT5.a-P2 AAGTGCCAGATAGTAGACGGTGAGAGGGTGTACAAAGCTGAGCCTATTTGTAAGTATTTCTTGAGATACAATATTGAAGAAATGGGGACACTTGCTTCTC 400

Clustal Consensus **************************************************************************************************** 399

BnaA.IGMT5.a-P1 AGTTCATCCTTGAACTTGATAGTGTCTTCCTCAACACATGGTAATAATTACTTTCTTGATTTATTTACAGTTTCTGTATATTCTCTTTATTTCATAATAT 500

BnaA.IGMT5.a-P2 AGTTCATCCTTGAACTTGATAGTGTCTTCCTCAACACATGGTAATAATTACTTTCTTGATTTATTTACAGTTTCTGTATATTCTCTTTATTTCATAATAT 500

Clustal Consensus **************************************************************************************************** 499

BnaA.IGMT5.a-P1 AAGTTGTTGAAGATTTTTTTCTTCTAAATATAAGTTATTTTCACATTTCATGCAGTTTTTTATTTATTGGATATTGTATGACCAACCAAATTATGCTGTT 600

BnaA.IGMT5.a-P2 AAGTTGTTGAAGATTTTTTTCTTCTAAATATAAGTTATTTTCACATTTCATGCAGTTTTTTATTTATTGGATATTGTATGACCAACCAAATTATGCTGTT 600

Clustal Consensus **************************************************************************************************** 598

BnaA.IGMT5.a-P1 TTTTATTTATTAAAATATATCTAGTTTATATCTTAAATTAAAAATTTAAAGTGTAATAACTTTCTTACATGTAAAATAATTTACATTATGAACACATAGG 700

BnaA.IGMT5.a-P2 TTTTATTTATTAAAATATATCTAGTTTATATCTTAAATTAAAAATTTAAAGTGTAATAACTTTCTTACATGTAAAATAATTTACATTATGAACACATAGG 700

Clustal Consensus **************************************************************************************************** 698

BnaA.IGMT5.a-P1 GAGTATAATTTACAAAAATGGTTTATTCTTTTACAGGGCACAGTTGAAAGATGTGGTGCTAGAAGGAGGAGATGCATTTGCTCGTGCCAACGGTGGGTTG 800

BnaA.IGMT5.a-P2 GAGTATAATTTACAAAAATGGTTTATTCTTTTACAGGGCACAGTTGAAAGATGTGGTGCTAGAAGGAGGAGATGCATTTGCTCGTGCCAACGGTGGGTTG 800

Clustal Consensus **************************************************************************************************** 798

BnaA.IGMT5.a-P1 AAGCTCTTTGATTACATGGGCACAGATGAAAGACTAAGCAAACTCTTTAACCGGACTGGATTCAGCGTTGGAGTTATGCAGAAGTTTCTTGAAGTTTATA 900

BnaA.IGMT5.a-P2 AAGCTCTTTGATTACATGGGCACAGATGAAAGACTAAGCAAACTCTTTAACCGGACTGGATTCAGCGTTGGAGTTATGCAGAAGTTTCTTGAAGTTTATA 900

Clustal Consensus **************************************************************************************************** 898

BnaA.IGMT5.a-P1 AAGGTTTTGAAGGAATCAATGTGTTGGTTGATGTAGGAGGAGGAGTTGGAAACACACTAGGTTTTGTTACTTCAAAGTATCCAAACATTAAGGGTATTAA 1000

BnaA.IGMT5.a-P2 AAGGTTTTGAAGGAATCAATGTGTTGGTTGATGTAGGAGGAGGAGTTGGAAACACACTAGGTTTTGTTACTTCAAAGTATCCAAACATTAAGGGTATTAA 1000

Clustal Consensus **************************************************************************************************** 996

BnaA.IGMT5.a-P1 TTTTGATCTAACTTGTGCTTTGGCACAAGCACCTTCTTATCCTAATGTGGAGCATGTGGCTGGAGATATGTTTGTAGAAATCCCAAGAGGAGATGCTATC 1100

BnaA.IGMT5.a-P2 TTTTGATCTAACTTGTGCTTTGGCACAAGCACCTTCTTATCCTAATGTGGAGCATGTGGCTGGAGATATGTTTGTAGAAATCCCAAGAGGAGATGCTATC 1100

Clustal Consensus **************************************************************************************************** 1096

BnaA.IGMT5.a-P1 ATCTTGAAAGTAAGACACAGCCGCAAACACTTACTCTTATCAAATATTTAATATGTTAATACTTCTCAAGTTTTTTTTATCCTTTATACTTACTTCATAT 1200

BnaA.IGMT5.a-P2 ATCTTGAAAGTAAGACACAGCCGCAAACACTTACTCTTATCAAATATTTAATATGTTAATACTTCTCAAGTTTTTTTTATCCTTTATACTTACTTCATAT 1200

Clustal Consensus **************************************************************************************************** 1196

BnaA.IGMT5.a-P1 GTTACTCCTGTATAGTGTGCTAATACTTCTTAAGGTGTTTTATTATTTATACTTACTTCATATATTACTCTTGTCTAATATGTTAATACTTCTCAGGTGT 1300

BnaA.IGMT5.a-P2 GTTACTCCTGTATAGTGTGCTAATACTTCTTAAGGTGTTTTATTATTTATACTTACTTCATATATTACTCTTGTCTAATATGTTAATACTTCTCAGGTGT 1300

Clustal Consensus **************************************************************************************************** 1296

BnaA.IGMT5.a-P1 TTTTTTTTTCTTTATTCTTACTTCATATGTTACTCTTGTCTAATATGTTAATACTTCTCAGGTGTTTTATTCTTTATATTTACTAACTTCATATATTTTA 1400

BnaA.IGMT5.a-P2 TTTTTTTTTCTTTATTCTTACTTCATATGTTACTCTTGTCTAATATGTTAATACTTCTCAGGTGTTTTATTCTTTATATTTACTAACTTCATATATTTTA 1400

Clustal Consensus **************************************************************************************************** 1395

BnaA.IGMT5.a-P1 CAGCGTATGCTTCATGATTGGAATGATGAAGACTGTGCAAAGATTCTCAAGAACTGCTGGAAGGCATTACCGGAGAATGGGAAAGTGATAATCATGGAGC 1500

BnaA.IGMT5.a-P2 CAGCGTATGCTTCATGATTGGAATGATGAAGACTGTGCAAAGATTCTCAAGAACTGCTGGAAGGCATTACCGGAGAATGGGAAAGTGATAATCATGGAGC 1500

Clustal Consensus **************************************************************************************************** 1494

BnaA.IGMT5.a-P1 TAGTTATTCCAGATGAGGCAGAGAGTAAAGATGTGCAGGCCAACATTGCATTTGATATGGATTTGTTGATGCTCACACAACTCTCTGGAGGAAAAGAGAG 1600

BnaA.IGMT5.a-P2 TAGTTATTCCAGATGAGGCAGAGAGTAAAGATGTGCAGGCCAACATTGCATTTGATATGGATTTGTTGATGCTCACACAACTCTCTGGAGGAAAAGAGAG 1600

Clustal Consensus **************************************************************************************************** 1594

BnaA.IGMT5.a-P1 AACTAAAGCTGAGTATGAAGCTATGGCTGCTAATTCAGGTTTTGCAAGTTGCAAATTTGTATGCCCTGCATATCATTTATGGGTCATTGAGTTCTCTAAA 1700

BnaA.IGMT5.a-P2 AACTAAAGCTGAGTATGAAGCTATGGCTGCTAATTCAGGTTTTGCAAGTTGCAAATTTGTATGCCCTGCATATCATTTATGGGTCATTGAGTTCTCTAAA 1700

Clustal Consensus **************************************************************************************************** 1694

BnaA.IGMT5.a-P1 TAG 1703

BnaA.IGMT5.a-P2 TAG 1703

Clustal Consensus *** 1697

***BnaA.IGMT5.b***

BnaA.IGMT5.b-P1 CTCAATACAAAATGTTCAAAAACTTGGTAGACGGTAACTTTATAGACCACTAACGCCAAGACAAATTATTTGGGGCCTAGTTGGAAGTGCAGACGCTAAC 100

BnaA.IGMT5.b-P2 CTCAATACAAAATGTTCAAAAACTTGGTAGACGGTAACTTTATAGACCACTAACGCCAAGACAAATTATTTGGGGCCTAGTTGGAAGTGCAGACGCTAAC 100

Clustal Consensus **************************************************************************************************** 100

BnaA.IGMT5.b-P1 TAATCATATATAATCTATTTACTATATTTTACATTTATATTAGATCTTTGTAATTTTGTGTTTAATTAAAACTTACTTTGATGAAATATAAAAATGAATA 200

BnaA.IGMT5.b-P2 TAATCATATATAATCTATTTACTATATTTTACATTTATATTAGATCTTTGTAATTTTGTGTTTAATTAAAACTTACTTTGATGAAATATAAAAATGAATA 200

Clustal Consensus **************************************************************************************************** 200

BnaA.IGMT5.b-P1 TACTAAAGAAAATCAAACAAACTTGTGAACTTTCAATAAGAAACAAAGTTGGAAATAAAAAAGATCATCACTAAATTAAAAAAAAAAATTGATGATAAAA 300

BnaA.IGMT5.b-P2 TACTAAAGAAAATCAAACAAACTTGTGAACTTTCAATAAGAAACAAAGTTGGAAATAAAAAAGATCATCACTAAATTAAAAAAAAAAATTGATGATAAAA 300

Clustal Consensus **************************************************************************************************** 300

BnaA.IGMT5.b-P1 ATTATATAATTTAATTAAACATAATCTGTTTTGATATTACTTGATTTTATCTATTTAGACCCATTTAAAACAATATAGACCGATTAAAAAATTTACTTAT 400

BnaA.IGMT5.b-P2 ATTATATAATTTAATTAAACATAATCTGTTTTGATATTACTTGATTTTATCTATTTAGACCCATTTAAAACAATATAGACCGATTAAAAAATTTACTTAT 400

Clustal Consensus **************************************************************************************************** 400

BnaA.IGMT5.b-P1 GATGATTTTACCGACTTGCTCAAACCAATTTTTATAATAGTGGAAGATACCAAAGAAGAGTTTCTTTGCTGAATAATGAAAACTTTCAGGGCACAACTGA 500

BnaA.IGMT5.b-P2 GATGATTTTACCGACTTGCTCAAACCAATTTTTATAATAGTGGAAGATACCAAAGAAGAGTTTCTTTGCTGAATAATGAAAACTTTCAGGGCACAACTGA 500

Clustal Consensus **************************************************************************************************** 500

BnaA.IGMT5.b-P1 AAGATGTGGTGCTAGAAGGAGGAGATGCATTTGCTCGTGCCAACGGTGGGTTGAAGCTCTTTGATTACATGGGCACAGATGAGAGACTAAGCAAACTCTT 600

BnaA.IGMT5.b-P2 AAGATGTGGTGCTAGAAGGAGGAGATGCATTTGCTCGTGCCAACGGTGGGTTGAAGCTCTTTGATTACATGGGCACAGATGAGAGACTAAGCAAACTCTT 600

Clustal Consensus **************************************************************************************************** 600

BnaA.IGMT5.b-P1 TAACAGGACTGGATTCAGCGTTGGGGTTTTACAGAAATTTCTAGAAGTGTACAAAGGCTTCGAAGGAGTTAATGTGTTGGTTGATGTAGGAGGAGGAGTT 700

BnaA.IGMT5.b-P2 TAACAGGACTGGATTCAGCGTTGGGGTTTTACAGAAATTTCTAGAAGTGTACAAAGGCTTCGAAGGAGTTAATGTGTTGGTTGATGTAGGAGGAGGAGTT 700

Clustal Consensus **************************************************************************************************** 700

BnaA.IGMT5.b-P1 GGAAACACACTAGGTTTTGTTACTTCAAAGTATCCAAACATTAAGGGTATCAACTTTGATCTAACTTGTGCTTTGACACAAGCACCTTCTTATCCTAATG 800

BnaA.IGMT5.b-P2 GGAAACACACTAGGTTTTGTTACTTCAAAGTATCCAAACATTAAGGGTATCAACTTTGATATAACTTGTGCTTTGACACAAGCACCTTCTTATCCTAATG 800

Clustal Consensus ************************************************************ *************************************** 799

BnaA.IGMT5.b-P1 TGGAGCATGTGGCTGGAGATATGTTTGTAGAAATCCCAAGAGGAGATGCTATCATCCTGAAAGTAAGACCAAACAAAAACTTCTTACTCTTGTCTATTTT 900

BnaA.IGMT5.b-P2 TGGAGCATGTGGCTGGAGATATGTTTGTAGAAATCCCAAGAGGAGATGCTATCATCCTGAAAGTAAGACCAAACAAAAACTTCTTACTCTTGTCTATTTT 900

Clustal Consensus **************************************************************************************************** 899

BnaA.IGMT5.b-P1 TCTGGTACTTACGTTCTTGATATGTTTTACAGCGTATGCTTCATGATTGGACTGATGAAGACTGTGCAAAGATTCTCAAGAATTGCTGGAAAGCGTTACC 1000

BnaA.IGMT5.b-P2 TCTGGTACTTACTTTCTTGATATGTTTTACAGCGTATGCTTCATGATTGGAGTGATGAAGACTGTGCAAAGATTCTCAAGAATTGCTGGAAAGCGTTACC 1000

Clustal Consensus ************ ************************************** ************************************************ 997

BnaA.IGMT5.b-P1 GGAGAATGGGAAAGTGATTATCATGGAACTAGTTATTCCAGATGAGGCAGAGAGTGCGGATGTGCAGTCCAACATTGCATTTGACATGGATTTGTTGATG 1100

BnaA.IGMT5.b-P2 GGAGAATGGGAAAGTGATTATCATGGAACTAGTTATTCCAGATGAGGCAGAGAGTGCGGATGTGCAGTCCAACATTGCATTTGACATGGATTTGTTGATG 1100

Clustal Consensus **************************************************************************************************** 1097

BnaA.IGMT5.b-P1 CTCACACAATGCTCTGGAGGAAAAGAGAGATCACGAGCTGAGTATGAAGCTATGGCTGCAAACTCGGGTTTTGCCAGTTGCCAGTTTGTATGCCAAGCTT 1200

BnaA.IGMT5.b-P2 CTCACCCAATGCTCTGGAGGAAAAGAGAGATCACGAGCTGAGTATGAAGCTATGGCTGCAAACTCGGGTTTCGCCAGTTGCCAGTTTGTATGCCAAGCTT 1200

Clustal Consensus ***** ***************************************************************** **************************** 1195

BnaA.IGMT5.b-P1 ATCATTTGTGGGTCATTGAGTTCTCTAAATAG 1232

BnaA.IGMT5.b-P2 ATCATTTGTGGGTCATTGAGTTCTCTAAATAG 1232

Clustal Consensus ******************************** 1227

***BnaC.IGMT5.b***

BnaC.IGMT5.b-P1 ATGGGATACGTTTCAGACCCTAAATCCATGAATGAGATTAATGGAGATGATGAGACCGAGCTTGGTTTGAGGGCGGTGAGGCTAGCCAATTACATAACCT 100

BnaC.IGMT5.b-P2 ATGGGATACGTTTCAGACCCTAAATCCATGAATGAGATTAATGGAGATGATGAGACCGAGCTTGGTTTGAGGGCGGTGAGGCTAGCCAATTACATAACCT 100

Clustal Consensus **************************************************************************************************** 100

BnaC.IGMT5.b-P1 TCCCAATGGTTTTCAAAGCCGCCATCGAACTTGGTGTCATCGATACTCTCTACTCAGCTGCTCGTGCTGATATGAATGGATCCAGTTCATTCCTCAAACC 200

BnaC.IGMT5.b-P2 TCCCAATGGTTTTCAAAGCCGCCATCGAACTTGGTGTCATCGATACTCTCTACTCAGCTGCTCGTGCTGATATGAATGGATCCAGTTCATTCCTCAAACC 200

Clustal Consensus **************************************************************************************************** 200

BnaC.IGMT5.b-P1 GTCTGAGATAGCTACTCGGCTTCCTACAACGCCTAGTAATCCTGAAGCACCTGCTTTGTTGGACCGTATGCTTCGTTTACTCGCTAGTTACTCAATGGTC 300

BnaC.IGMT5.b-P2 GTCTGAGATAGCTACTCGGCTTCCTACAACGCCTAGTAATCCTGAAGCACCTGCTTTGTTGGACCGTATGCTTCGTTTACTCGCTAGTTACTCAATGGTC 300

Clustal Consensus **************************************************************************************************** 300

BnaC.IGMT5.b-P1 AAATGCCAAATCCTAGATGGTGAGAGGGTTTACAAAGCTGAACCCATTTGCAAGTATTTCTTGAGATACAATATTGAAGAAATAGGAACACTTGCTTCTC 400

BnaC.IGMT5.b-P2 AAATGCCAAATCCTAGATGGTGAGAGGGTTTACAAAGCTGAACCCATTTGCAAGTATTTCTTGAGATACAATATTGAAGAAATAGGAACACTTGCTTCTC 400

Clustal Consensus **************************************************************************************************** 400

BnaC.IGMT5.b-P1 AATTCATTCTTGAACTCGACAGTGTCTTCCTCAATACATGGTAATTACTTTCTTGATCTCTTTACAACTCGATACAAAATGTTCAAAAAATTGTTTGACG 500

BnaC.IGMT5.b-P2 AATTCATTCTTGAACTCGACAGTGTCTTCCTCAATACATGGTAATTACTTTCTTGATCTCTTTACAACTCGATACAAAATGTTCAAAAAATTGTTTGACG 500

Clustal Consensus **************************************************************************************************** 500

BnaC.IGMT5.b-P1 GTAACTTTATAGACGACTAACGCCAAGACAAATTATTTGGGGCCTAGTTGGAAGTGCAGACGTTAACGAATTATATATCTATTTACTATATATTTTATAT 600

BnaC.IGMT5.b-P2 GTAACTTTATAGACGACTAACGCCAAGACAAATTATTTGGGGCCTAGTTGGAAGTGCAGACGTTAACGAATTATATATCTATTTACTATATATTTTATAT 600

Clustal Consensus **************************************************************************************************** 600

BnaC.IGMT5.b-P1 TTATATTAGATCTTTGTAATTTTGTGTTTAATTAAAGTTACTTTGATGAAATATAAAAATGAATATACTAAAAAATCAAACAAACTTGTGATCTTTCAAT 700

BnaC.IGMT5.b-P2 TTATATTAGATCTTTGTAATTTTGTGTTTAATTAAAGTTACTTTGATGAAATATAAAAATGAATATACTAAAAAATCAAACAAACTTGTGATCTTTCAAT 700

Clustal Consensus **************************************************************************************************** 700

BnaC.IGMT5.b-P1 AAGAAACAAAGTTGGAAAGAAAAAAAATCATCACTAAATTTTTAAAAATTATATAATTAAATTAACATAATCTATTTTGATATTACTTGATTTAACCTAT 800

BnaC.IGMT5.b-P2 AAGAAACAAAGTTGGAAAGAAAAAAAATCATCACTAAATTTTTAAAAATTATATAATTAAATTAACATAATCTATTTTGATATTACTTGATTTAACCTAT 800

Clustal Consensus **************************************************************************************************** 800

BnaC.IGMT5.b-P1 CTAGACCCATTTAAAATAATTTAGACCGATTAAATTTTATTTATGATGATTTTACCGACTTGCTCAAACCAATTTATATAATATTGGAAGATACCAAAGA 900

BnaC.IGMT5.b-P2 CTAGACCCATTTAAAATAATTTAGACCGATTAAATTTTATTTATGATGATTTTACCGACTTGCTCAAACCAATTTATATAATATTGGAAGATACCAAAGA 900

Clustal Consensus **************************************************************************************************** 900

BnaC.IGMT5.b-P1 AGAGTTTCTTTGCTTAGTAGTGAAAACTTTCAGGGCACAACTGAAAGATGTGGTGCTAGAAGGAGGAGATGCATTTGCTCGTGCCAACGGTGGGTTGAAG 1000

BnaC.IGMT5.b-P2 AGAGTTTCTTTGCTTAGTAGTGAAAACTTTCAGGGCACAACTGAAAGATGTGGTGCTAGAAGGAGGAGATGCATTTGCTCGTGCCAACGGTGGGTTGAAG 1000

Clustal Consensus **************************************************************************************************** 1000

BnaC.IGMT5.b-P1 CTCTTTGATTACATGGGAACGGATGAGAGACTAAGCAAACTCTTTAACCGGACTGGATTCAGCGTTGGGGTTTTGCAGAAGTTTCTAGAAGTTTACAAAG 1100

BnaC.IGMT5.b-P2 CTCTTTGATTACATGGGAACGGATGAGAGACTAAGCAAACTCTTTAACCGGACTGGATTCAGCGTTGGGGTTTTGCAGAAGTTTCTAGAAGTTTACAAAG 1100

Clustal Consensus **************************************************************************************************** 1100

BnaC.IGMT5.b-P1 GTTTCGAAGGAGTTAATGTGTTGGTTGATGTAGGAGGAGGAGTTGGAAACACACTAGGCTTTGTTACTTCAAAGTATCCAAACATTAAGGGTATTAATTT 1200

BnaC.IGMT5.b-P2 GTTTCGAAGGAGTTAATGTGTTGGTTGATGTAGGAGGAGGAGTTGGAAACACACTAGGCTTTGTTACTTCAAAGTATCCAAACATTAAGGGTATTAATTT 1200

Clustal Consensus **************************************************************************************************** 1200

BnaC.IGMT5.b-P1 TGATCTAACTTGTGCTTTGACACAAGCACCTTCTTATCCTAATGTGGAGCATGTGGCTGGAGATATGTTTGTAGAAGTCCCAAGAGGAGATGCTATCATC 1300

BnaC.IGMT5.b-P2 TGATCTAACTTGTGCTTTGACACAAGCACCTTCTTATCCTAATGTGGAGCATGTGGCTGGAGATATGTTTGTAGAAGTCCCAAGAGGAGATGCTATCATC 1300

Clustal Consensus **************************************************************************************************** 1300

BnaC.IGMT5.b-P1 TTGAAAGTAAGACTAAACAATAAACTCCTACTCGTCTTTAATATGTTAATACTTCTCATCTATGTGTTTTACTTACTTGATATGTTTTATAGCGTATGCT 1400

BnaC.IGMT5.b-P2 TTGAAAGTAAGACTAAACAATAAACTCCTACTCGTCTTTAATATGTTAATACTTCTCATCTATGTGTTTTACTTACTTGATATGTTTTATAGCGTATGCT 1400

Clustal Consensus **************************************************************************************************** 1400

BnaC.IGMT5.b-P1 TCATGATTGGAGTGATGAAGACTGTGCAAAGATTCTCAAGAATTGCTGGAAAGCGTTACCGGAGAATGGGAAAGTGATTATCATGGAACTAGTAATTCCA 1500

BnaC.IGMT5.b-P2 TCATGATTGGAGTGATGAAGACTGTGCAAAGATTCTCAAGAATTGCTGGAAAGCGTTACCGGAGAATGGGAAAGTGATTATCATGGAACTAGTAATTCCA 1500

Clustal Consensus **************************************************************************************************** 1500

BnaC.IGMT5.b-P1 GATGAGGCAGAGAGTGCAGATGTGCAGTCCAACATTGCATTTGACATGGATTTGTTGATGCTCACACAATGCTCTGGAGGAAAAGAGAGATCAAGGGCTG 1600

BnaC.IGMT5.b-P2 GATGAGGCAGAGAGTGCAGATGTGCAGTCCAACATTGCATTTGACATGGATTTGTTGATGCTCACACAATGCTCTGGAGGAAAAGAGAGATCAAGGGCTG 1600

Clustal Consensus **************************************************************************************************** 1600

BnaC.IGMT5.b-P1 AGTATGAAGCTATGGCTGCAGATTCGGGTTTTGCCAATTGCAAGTTTGTATGCCAAGCTTATCATTTGTGGGTCATTGAGTTCACTAAATAG 1692

BnaC.IGMT5.b-P2 AGTATGAAGCTATGGCTGCAGATTCGGGTTTTGCCAATTGCAAGTTTGTATGCCAAGCTTATCATTTGTGGGTCATTGAGTTCACTAAATAG 1692

Clustal Consensus ******************************************************************************************** 1692

***BnaC.IGMT5.a***

BnaC.IGMT5.a-P1

ATGGGACACCTTGTAGACCCTAAAACCATGAATGAGATTAATGGAGATGATGAGACCGAGCTTGGTTTGAGGGCAGTGAGGCTAGCCAATTACATTACCT 100

TCCCCATGGTTTTCAAAGCCGCCATTGAGCTCGGTGTCATCGACGCTCTCTACTTAGCTGCTCGTGATGACGTCAATGGATCTGGATCGTTCCTCAAACC 200

GTCTGAGATAGCTACTCGGCTTCCCACACCGCCTAGTAACCCTGAAGCACCGGTTTTACTGGACCGTATGCTTCGTTTACTCGCCAGTTACTCAATGGTC 300

AAGTGTCAGATAGTAGACGGCGAGAGGGTGTACAAAGCTGAGCCCATTTGTAAGTATTTCTTGAGATACAATATTGAAGAAATGGGGACACTTGCTTCTC 400

AGTTCATTCTTGAACTTGATAGTGTCTTCCTCAACACATGGTAATAATTACTTTCTTGATTTATTTACAGTTTCTGTATTCTCTTTTTTATTTCATAATC 500

TAAGTTGTTGAATTTTTTTTCTTCTAAATATTAGTTATTTTCACATTTTATGCAATTTTTTATTTATTGGTTATTGTACGACCAACCAAATTATGCTGTT 600

TTTTATTTATTAAAATATATCTAGTTTATATCTTAAATTAAAATTTTAAAGTGTAATAACTTTATTACATGTAAAATAATTTACATTATGAAACATAGGG 700

AGTATAATTTACAAAAATGGTTTATTCTTTTACAAACTTTTTCAGGGCACAGTTGAAAGATGTGGTGCTAGAGGGAGGAGATGCATTTGCTCGTGCCAAT 800

GGTGGGTTGAAGCTCTTTGATTACATGGGCACAGATGAAAGACTAAGCAAACTCTTTAACCGGACTGGATTCAGCGTTGGAGTTATGCAGAAGTTTCTTG 900

AAGTTTATAAAGGTTTCGAAGGAATCAATGTGTTGGTTGATGTAGGAGGAGGAGTTGGAAACACACTAGGTTTTGTTACTTCAAAGTATCCAAACATTAA 1000

GGGTATTAATTTTGATCTAACTTGTGCTTTGGCACAAGCACCTTCTTATCCTAATGTGGAACATGTGGCTGGAGATATGTTTGTAGAAATCCCAAGAGGA 1100

GATGCTATCATCTTGAAAGTAAGACACAGCCACAAACACTTACTCTTGTCTAATATATTAATACTTCTCAAGTTTTTTTATCCTTTATACTTACTTCATA 1200

TGTTACTCCTGTCTAGTGTGCTAATACTTCTTAAGGTGTTTTATTATTTATACTTGCTTCATATATTAATCTTGTCTAATATGTTAATACTTCTCAGGTG 1300

TTTTTTTTTTCTTTATTCTTACTTCATATGTTACTCTTGTCTAATATGTTAATACTTCTCAGGTGTTTTATTCTTTATATTTACTTACTTCATATGTTTT 1400

CCAGCGTATGCTTCATGATTGGAATGATGAAGACTGTGCAAAGATTCTCAAGAACTGCTGGAAGGCATTACCGGAGAATGGGAAAGTGATAATCATGGAG 1500

CTAGTTATTCCAGATGAGGCAGAGAGTAAAGATGTGCAGGCCAACATTGCATTTGATATGGATTTGTTGATGCTCACACAACTCTCTGGAGGAAAAGAGA 1600

GAACAAAAGCTGAGTATGAAGCTATGGCTGCTAATTCAGGTTTTGCAAGTTGCAAATTTGTGTGCCCTGCATATCATTTATGGGTCATTGAGTTCTCTAA 1700

ATAG 1704

**Figure S3** Alignment of the genomic nucleotide sequences of all copies of *IGMT5* between Hua 5 (P1) and J7005 (P2).
