# Supplementary material for: Identification of QTLs for Resistance to Sclerotinia Stem Rot and BnaC.IGMT5.a as a Candidate Gene of the Major Resistant QTL SRC6 in Brassica napus
Source: PLoS One. 2013 Jul 2;8(7):e67740. doi: 10.1371/journal.pone.0067740 (PMC3699613; doi:10.1371/journal.pone.0067740)
Supplement: Figure S4 — Alignment of the genomic nucleotide sequences of BraA.IGMT5.b and BnaA.IGMT5.b, and the deletion in BnaA.IGMT5.b. (DOCX) [file pone.0067740.s004.docx]

BraA.IGMT5.b GCTATAACATTAGCAACTAGGTACGCTGATTAAATAAAAATCAATGGAACCATACTTTAAGTGGTTTATTGAATAAAATGTAGAGTATAAGATTTAAACT 100

BnaA.IGMT5.b GCTATAACATTAGCAACTAGGTACGCTGATTAAATAAAAATCAATGGAACCATACTTTAAGTGGTTTATTGAATAAAATGTAGAGTATAAGATTTAAACT 100

Clustal Consensus **************************************************************************************************** 100

BraA.IGMT5.b TTTTATGTTTATAAAAACATCTTAACCTTTTCATTTTCAAAAAAAAAAC--AAAAAAAAAATATCTTAAACTTTTACTTGTTTAGAATCTTCAGGCATAA 198

BnaA.IGMT5.b TTTTATGTTTATAAAAACATCTTAAACTTTTCATTTTCAAAAAAAAAAACAAAAAAAAAAATATCTTAAACTTTTACTTGTTTAGAATCTTTAGGCATAA 200

Clustal Consensus ************************* ********************** **************************************** ******** 195

BraA.IGMT5.b GCATGTGCTCGGAATTTTTAGCTTGTTCAGAAAATCTTAACTATTTTGAATTGCCACTCTAACTCAGTAAGTGGCCAGCACGACAATATACAATGAACAA 298

BnaA.IGMT5.b GCATGTGCTCGGAATTTTTAGCTTGTTCAGAAAATCTTAACTATTTTGAATTGCCACTCTAACTCAGTAAGTGGTCAGCACGACAATATACAATGAACAA 300

Clustal Consensus ************************************************************************** ************************* 294

BraA.IGMT5.b TTCAAAAATTATCAGCACGAAAGGTCCCACAGATATATAGGTACTTGGTTCGAGATCTTAAACTAAAATTTAGTTGGATGACAAAAAAAAAAACTAAAAT 398

BnaA.IGMT5.b TTCAAAAATTATCAGCACGAAAGGTCCCACAGATATATAGGTACTTGGTTCGAGATCTTAAACTAAAATTTAGTTGGATGACAAAAAAAA--ACTAAAAT 398

Clustal Consensus ****************************************************************************************** ******** 392

BraA.IGMT5.b TTAGTTAATCCAAACATTTAAGTGAATATAAATTATAATAAAGTGAGTATATGAATTGATTGAACATTACAAGAATAATATACAGAATGAAATTGTTTTA 498

BnaA.IGMT5.b TTAGTTAATCCAAACATTTAAGTGAATATAAATTATAATAAAGTGAGTATATGAATTGATTGAACATTACAAGAATAATATACAGAATGAAATTGTTTTA 498

Clustal Consensus **************************************************************************************************** 492

BraA.IGMT5.b TTTAGAGCCTGTATGCCCTCTACTGCTCTACATATAACTGAATATAATATTTCGCCAATATACATATTTTAGAGCAATTTTATTGGTAGTCTCTAACAAA 598

BnaA.IGMT5.b TTTAGAGCCTGTATGCCCTCTACTGCTCTACATATAACTGAATATAATATTTCGCCAATATACATATTTTAGAGCAATTTTATTGGTAGTCTCTAACAAA 598

Clustal Consensus **************************************************************************************************** 592

BraA.IGMT5.b GTTTCTAGACATTAAAAAGTAAAAAAAAAAGTAGAGAGAGAATGAAAAAATTGTAGAAAGAAAAGGAATGAATTTGTCGAGGGATTTACTGTAGTGAAAA 698

BnaA.IGMT5.b GTTTCTAGACATTAAAAAGTAAAAAAAAAAGTAGAGAGAGAATGAAAAAATTGTAGAAAGAAAAGGAATGAATTTGTCGAGGGATTTACTGTAGTGAAAA 698

Clustal Consensus **************************************************************************************************** 692

BraA.IGMT5.b GCTATATTGACACTTGCTTCCAACGAGCTATTACTTTGATTTTAACATTGTTTGATTAACTATGGTTTTGAATGGTGACGGACCGCACAATAACACAAAT 798

BnaA.IGMT5.b GCTATATTGACACTTGCTTCCAACGAGCTATTACTTTGATTTTAACATTGTTTGATTAACTATGGTTTTGAATGGTGACGGACCGCACAATAACACAAAT 798

Clustal Consensus **************************************************************************************************** 792

BraA.IGMT5.b CTCTACATATATATTTATATGTTTTTGTTACTATTCAAAGCGCACCGCAACTATTCCATGGGTGTACTCCTGCACCTACGTTACCATTCAGAGACATATG 898

BnaA.IGMT5.b CTCTACATATATATTTATATGTTTTTGTTACTATTCAA-------------------------------------------------------------- 836

Clustal Consensus ************************************** 830

BraA.IGMT5.b AATTAACGTGTCCAAATCCCTGTCATGATCTTGATCTTGTTGAGGGATCATTTGCATCGTTAGAGTGAGAAACCATATTGACACTAGTGGTGCACAGGTA 998

BnaA.IGMT5.b ---------------------------------------------------------------------------------------------------- 836

Clustal Consensus 830

BraA.IGMT5.b CGTTTAGGGGTGGGCGTTCGGGTACCCGTTCGAGTTCGGGTCGGGTATTTCGGATTTTCGGGTATTTCGGTATAGAGGTCTAGAATCCGTTTTCATTTCT 1098

BnaA.IGMT5.b ---------------------------------------------------------------------------------------------------- 836

Clustal Consensus 830

BraA.IGMT5.b CAAGTTTTTTTATTTAAAAATATAACTTTTAGTTAACTAATTTTTTATTTTTAATAGATTGAATGGTTAATAGATTTGGACATAACATTTTAAAACTAAA 1198

BnaA.IGMT5.b ---------------------------------------------------------------------------------------------------- 836

Clustal Consensus 830

BraA.IGMT5.b AAGGCACTAATTTAGTTATTTTTTTTAATTTTGGATATAACTTTTTGTTAATTTTTGAAATAAAAAACTTGACATGCATTTTAAGTGAGTAGCAAATCAT 1298

BnaA.IGMT5.b ---------------------------------------------------------------------------------------------------- 836

Clustal Consensus 830

BraA.IGMT5.b TTTTCCGTAATTGTATGTATATTATATGAACTTAAATTATGTGTAGTATCAATATAAATATTTTATATAAAATGAGAGATATAAACTAGAAACATAAGGT 1398

BnaA.IGMT5.b ---------------------------------------------------------------------------------------------------- 836

Clustal Consensus 830

BraA.IGMT5.b TAATTATACATATGTTCGGTTATCTTCGGATATCCATTCGGGTTCGGGTATTATTCGTTCGGGTTCGGGTATCCAATCTCTCCTTATTCAATACCCGTTC 1498

BnaA.IGMT5.b ---------------------------------------------------------------------------------------------------- 836

Clustal Consensus 830

BraA.IGMT5.b GGGTATTTTGCTACTTCGGTTCGGATTTCGGTTCGGTTTTTTCGGATCGGGTTCGGGTGCCACTTCGGATATCGGGTAAAGTGCCCACCCCTAGGTACGT 1598

BnaA.IGMT5.b ---------------------------------------------------------------------------------------------------- 836

Clustal Consensus 830

BraA.IGMT5.b TGACAGTATATTTGGATCCATTCTCCACCTACCATTCTCCATAGTCTTTTTTTAATTCCTCACATTTTCTTTTACTAATTTCCATTTTTTTTTTATAAAA 1698

BnaA.IGMT5.b ---------------------------------------------------------------------------------------------------- 836

Clustal Consensus 830

BraA.IGMT5.b CCAATTCCACCTAACTTTTTTTATTAATTATGCCCACATGATGTGTTATGAACCTTTTTCCAACCAATTTAATAAGAAATTCTCCAGTTCCAACAAAATG 1798

BnaA.IGMT5.b ---------------------------------------------------------------------------------------------------- 836

Clustal Consensus 830

BraA.IGMT5.b ATGTTAGGTCATGTCCATTATTAAAATGAAAAAGTATCTAAAAATTATATGATATAAAATAATAAAATTGGGTATATAAAGTATGTATCTAAAAATCTAT 1898

BnaA.IGMT5.b ---------------------------------------------------------------------------------------------------- 836

Clustal Consensus 830

BraA.IGMT5.b ACACTTTCTAGTTTTAAAATACTCATTTTATATTTTATAGTTATTTAGTATTATATAAATTTTAAGATATCCACCCTCATTCTTGCGTGTTAAAAAAGTT 1998

BnaA.IGMT5.b ---------------------------------------------------------------------------------------------------- 836

Clustal Consensus 830

BraA.IGMT5.b CGAGTACAAGTTCAGAAAAAAAAAAAAAAAGTTCGAGTACAAGTTGAATGCCAAAATGTTCAGTTATACACCCTAATGTTTTTGCGTGTTTCACGTGGCT 2098

BnaA.IGMT5.b ---------------------------------------------------------------------------------------------------- 836

Clustal Consensus 830

BraA.IGMT5.b GATTGTTTTAAAGGAATATATTTTGAAAACATCTACTCCCTCCGTTTCGTTATGTAAGTAGTTTTGCTTAAAAGCACGGCTATTAAGAAACTTCAACTTT 2198

BnaA.IGMT5.b ---------------------------------------------------------------------------------------------------- 836

Clustal Consensus 830

BraA.IGMT5.b TTCTAAAACAACAATAAAAACGTTTTTGTATTTATTGCACACGTTCATTTTGTCTGCAACATTTAATTAGAAAGAGATTATTGTTAGTAATAAAATTAAT 2298

BnaA.IGMT5.b ---------------------------------------------------------------------------------------------------- 836

Clustal Consensus 830

BraA.IGMT5.b AAAAAATAATATCAACAATCACACGTTTGAGTTTTCTAAAAACTTTTTTACAACATTTTGGAGCCAAATTTTTGTATAACTAATTTAAACTAAAAAATCA 2398

BnaA.IGMT5.b ---------------------------------------------------------------------------------------------------- 836

Clustal Consensus 830

BraA.IGMT5.b AAATTTTATGATGGAGCCAAAGTAAAACTTTTGAACAGTTTGTAATATGCTTATTTAAGCTTCAATTTCTTTGTCGGGATGAAGCTCTCCAATATCATAA 2498

BnaA.IGMT5.b ---------------------------------------------------------------------------------------------------- 836

Clustal Consensus 830

BraA.IGMT5.b TGCATCTTGTTCAAGTCAAATGGCCGAGTGAACATCTGGAGGTTTGATTGTGTTTTGTTGATTTTTTTAACATTTTCTGTTTTGTATTCTTTTTAACTTT 2598

BnaA.IGMT5.b ---------------------------------------------------------------------------------------------------- 836

Clustal Consensus 830

BraA.IGMT5.b TTTCTTCATTTTGATCCATAGTTTTATGAAGAAAAAATAGATCGAAGAAAGTAAATCAAAAAGAAAAACTAATGGAGTAAGACAACATTTACGGTGGTAT 2698

BnaA.IGMT5.b ---------------------------------------------------------------------------------------------------- 836

Clustal Consensus 830

BraA.IGMT5.b TTATAGTGAATTTAGAAATAGATTTTTTGGTAACTAGTAGTAAATGTTAGTTGACTGACATTGTAAGTTGTGTTTAAAAAATTGAAAACTAACGACAAGG 2798

BnaA.IGMT5.b ---------------------------------------------------------------------------------------------------- 836

Clustal Consensus 830

BraA.IGMT5.b CCTTTAAGTTTTTTAGTGGAATATTGTATATTTATTGTTTTGGGTTCCGAGAACAACTTATATTATGAAACAAATATTTTTTGGCAAAACTGCTTACATA 2898

BnaA.IGMT5.b ---------------------------------------------------------------------------------------------------- 836

Clustal Consensus 830

BraA.IGMT5.b ACGAAACGGAGGGAGTAGTAGATAGCGTCACAGTGCTAGGCGCTTCAGATGGAACTCCCTCAAAAATCACGTGATCGCCGACACAATTATTATTATTATT 2998

BnaA.IGMT5.b ---------------------------------------------------------------------------------------------------- 836

Clustal Consensus 830

BraA.IGMT5.b ATTATTGCTTATACTTACCAATTCGACGCCTACAAAAGGAACCACAAAAATAGAAGAAAAAAGACATATGCAATAAAAAGATCTTCGTCTTCTCTTTCTT 3098

BnaA.IGMT5.b ---------------------------------------------------------------------------------------------------- 836

Clustal Consensus 830

BraA.IGMT5.b TTGATCTAAACACATTGCTTTGACGTAGAAGAAAGCAACAAAAAATGGGATACGTTTCAGACCCTAAATCCATGAATGAGATTAATGGAGATGATGAGAC 3198

BnaA.IGMT5.b ---------------------------------------------------------------------------------------------------- 836

Clustal Consensus 830

BraA.IGMT5.b CGAGCTTGGTTTGAGGGCGGTGAGGCTAGCCAATTACATAACCTTCCCAATGGTTTTCAAAGCCGCCATCGAACTTGGTGTCATCGATACTCTCTACGCA 3298

BnaA.IGMT5.b ---------------------------------------------------------------------------------------------------- 836

Clustal Consensus 830

BraA.IGMT5.b GCTGCTCGTGCTGATGTCAATGGATCCAGTTCATTCCTCAAACCGTCTGAGATAGCTACTCGGCTTCCTACAACGCCTAGTAATCCTGAAGCACCTGCTT 3398

BnaA.IGMT5.b ---------------------------------------------------------------------------------------------------- 836

Clustal Consensus 830

BraA.IGMT5.b TGTTGGACCGTATGCTTCGTTTACTCGCTAGTTACTCAATGGTCAAATGCCAAATCCTAGATGGTGAGAGGGTTTACAAAGCTGAACCCATTTGTAAGTA 3498

BnaA.IGMT5.b ---------------------------------------------------------------------------------------------------- 836

Clustal Consensus 830

BraA.IGMT5.b TTTCTTGAGATACAATATTGAAGAAATGGGAACACTTGCTTCTCAATTCATTCTTGAACTCGACAGTGTCTTCCTGAATACATGGTAATTACTTTCTTGA 3598

BnaA.IGMT5.b ---------------------------------------------------------------------------------------------------- 836

Clustal Consensus 830

BraA.IGMT5.b TCTCTTCTCAACTCAATACAAAATGTTCAAAAAATTGGTAGACGGTAACTTTATAGACCACTAACGCCAAGACAAATTATTTGGGGCCTAGTTGGAAGTG 3698

BnaA.IGMT5.b -----------CTCAATACAAAATGTTCAAAAACTTGGTAGACGGTAACTTTATAGACCACTAACGCCAAGACAAATTATTTGGGGCCTAGTTGGAAGTG 925

Clustal Consensus ********************** ****************************************************************** 918

BraA.IGMT5.b CAGACGTTAACTAATCATATATAATCTATTTACTATATTTTACATTTATATTAGATCTTTGTAATTTTGTGTTTAATTAAAACTTACTTTGATGAAATAT 3798

BnaA.IGMT5.b CAGACGCTAACTAATCATATATAATCTATTTACTATATTTTACATTTATATTAGATCTTTGTAATTTTGTGTTTAATTAAAACTTACTTTGATGAAATAT 1025

Clustal Consensus ****** ********************************************************************************************* 1017

BraA.IGMT5.b AAAAATGAATATACTAAAGAAAATCAAACAAACTTGTGAACTTTCAATAAGAAACAAAGTTGGAAATAAAAAAGATCATCACTAAATTAAAAAAAAAAAA 3898

BnaA.IGMT5.b AAAAATGAATATACTAAAGAAAATCAAACAAACTTGTGAACTTTCAATAAGAAACAAAGTTGGAAATAAAAAAGATCATCACTAAATTAAAAAAAAAAA- 1124

Clustal Consensus *************************************************************************************************** 1116

BraA.IGMT5.b TTGATGATAAAAATTATATAATTTAATTAAACATAATCTGTTTTGATATTACTTGATTTTATCTATTTAGACCCATTTAAAACAATATAGACCACCGATT 3998

BnaA.IGMT5.b TTGATGATAAAAATTATATAATTTAATTAAACATAATCTGTTTTGATATTACTTGATTTTATCTATTTAGACCCATTTAAAACAATATAGAC---CGATT 1221

Clustal Consensus ******************************************************************************************** ***** 1213

BraA.IGMT5.b AAAAAATTTACTTATGATGATTTTACCGACTTGCTCAAACCAATTTTTATAATAGTGGAAGATACCAAAGAAGAGTTTCTTTGCTGAATAATGAAAACTT 4098

BnaA.IGMT5.b AAAAAATTTACTTATGATGATTTTACCGACTTGCTCAAACCAATTTTTATAATAGTGGAAGATACCAAAGAAGAGTTTCTTTGCTGAATAATGAAAACTT 1321

Clustal Consensus **************************************************************************************************** 1313

BraA.IGMT5.b TCAGGGCACAACTGAAAGATGTGGTGCTAGAAGGAGGAGATGCATTTGCTCGTGCCAACGGTGGGTTGAAGCTCTTTGATTACATGGGCACAGATGAGAG 4198

BnaA.IGMT5.b TCAGGGCACAACTGAAAGATGTGGTGCTAGAAGGAGGAGATGCATTTGCTCGTGCCAACGGTGGGTTGAAGCTCTTTGATTACATGGGCACAGATGAGAG 1421

Clustal Consensus **************************************************************************************************** 1413

BraA.IGMT5.b ACTAAGCAAACTCTTTAACAGGACTGGATTCAGCGTTGGGGTTTTACAGAAATTTCTAGAAGTGTACAAAGGCTTCGAAGGAGTTAATGTGTTGGTTGAT 4298

BnaA.IGMT5.b ACTAAGCAAACTCTTTAACAGGACTGGATTCAGCGTTGGGGTTTTACAGAAATTTCTAGAAGTGTACAAAGGCTTCGAAGGAGTTAATGTGTTGGTTGAT 1521

Clustal Consensus **************************************************************************************************** 1513

BraA.IGMT5.b GTAGGAGGAGGAGTTGGAAACACACTAGGTTTTGTTACTTCAAAGTATCCAAACATTAAGGGTATCAACTTTGATCTAACTTGTGCTTTGACACAAGCAC 4398

BnaA.IGMT5.b GTAGGAGGAGGAGTTGGAAACACACTAGGTTTTGTTACTTCAAAGTATCCAAACATTAAGGGTATCAACTTTGATCTAACTTGTGCTTTGACACAAGCAC 1621

Clustal Consensus **************************************************************************************************** 1613

BraA.IGMT5.b CTTCTTATCCTAATGTGGAGCATGTGGCTGGAGATATGTTTGTAGAAAT----------GATGCTATCATCCTGAAAGTAAGACCAAACAAAAACTTCTT 4488

BnaA.IGMT5.b CTTCTTATCCTAATGTGGAGCATGTGGCTGGAGATATGTTTGTAGAAATCCCAAGAGGAGATGCTATCATCCTGAAAGTAAGACCAAACAAAAACTTCTT 1721

Clustal Consensus ************************************************* ***************************************** 1703

BraA.IGMT5.b ACTCTTGTCTATTTTTCTGGTACTTACTTTCTTGATATGTTTTACAGCGTATGCTTCATGATTGGAGTGATGAAGACTGTGCAAAGATTCTCAAGAATTG 4588

BnaA.IGMT5.b ACTCTTGTCTATTTTTCTGGTACTTACGTTCTTGATATGTTTTACAGCGTATGCTTCATGATTGGACTGATGAAGACTGTGCAAAGATTCTCAAGAATTG 1821

Clustal Consensus *************************** ************************************** ********************************* 1801

BraA.IGMT5.b CTGGAAAGCGTTACCGGAGAATGGGAAAGTGATTATCATGGAACTAGTTATTCCAGATGAGGCAGAGAGTGCGGATGTGCAGTCCAACATTGCATTTGAC 4688

BnaA.IGMT5.b CTGGAAAGCGTTACCGGAGAATGGGAAAGTGATTATCATGGAACTAGTTATTCCAGATGAGGCAGAGAGTGCGGATGTGCAGTCCAACATTGCATTTGAC 1921

Clustal Consensus **************************************************************************************************** 1901

BraA.IGMT5.b ATGGATTTGTTGATGCTCACCCAATGCTCTGGAGGAAAAGAGAGATCACGAGCTGAGTATGAAGCTATGGCTGCAAACTCGGGTTTCGCCAGTTGCCAGT 4788

BnaA.IGMT5.b ATGGATTTGTTGATGCTCACACAATGCTCTGGAGGAAAAGAGAGATCACGAGCTGAGTATGAAGCTATGGCTGCAAACTCGGGTTTTGCCAGTTGCCAGT 2021

Clustal Consensus ******************** ***************************************************************** ************* 1999

BraA.IGMT5.b TTGTATGCCAAGCTTATCATTTGTGGGTCATTGAGTTCTCTAAATAG 4835

BnaA.IGMT5.b TTGTATGCCAAGCTTATCATTTGTGGGTCATTGAGTTCTCTAAATAG 2068

Clustal Consensus *********************************************** 2046

**Figure S4** Alignment of the genomic nucleotide sequences of *BraA.IGMT5.b* and *BnaA.IGMT5.b*, and the deletion in *BnaA.IGMT5.b*.
