# Supplementary material for: Identification of QTLs for Resistance to Sclerotinia Stem Rot and BnaC.IGMT5.a as a Candidate Gene of the Major Resistant QTL SRC6 in Brassica napus
Source: PLoS One. 2013 Jul 2;8(7):e67740. doi: 10.1371/journal.pone.0067740 (PMC3699613; doi:10.1371/journal.pone.0067740)
Supplement: Table S1 — Primer sequences of the newly developed SSR markers in the study. (DOCX) [file pone.0067740.s005.docx]

**Table S1** Primer sequences of the newly developed SSR markers in the study.

| **Marker Name** | **Forward primer sequence(5'to3')** | **Reverse primer sequence(5'to3')** |
| --- | --- | --- |
| BEN10 | AACAGCAGAGAAGCGAGGAG | GAAGAGGGATCGGAAGAAGG |
| BEN119 | ATGGACAGGAGATGGTGGAG | GGTATCCACGGCTTGTGACT |
| BEN123 | AAAGACAAAGACTTGCGCGT | CTCCCACTCAGACTTCTCGG |
| BEN133 | TTGGGTGGTAAGAAAGATGGA | ACTTGCTCGTGTGGAAGACC |
| BEN136 | TGCGAGGATCCCAAGTTTAC | CAAACTGTTTCCTTGTGGCA |
| BEN14 | CAAGAGATCGTAGCTTCCGC | CAGTTGGCCAGAAGTGCATA |
| BEN140 | ACAGGACACGATACGGGAGA | GGCTTGCTTCAGTCCGTAAG |
| BEN16 | CTGGTCCAATGAGCAAACCT | GGAGGTGGTCCTTTCTTTCC |
| BEN185 | GGAGGAAGGGAACAGTGTGA | TCCGGAAGTGAAGTAATCGG |
| BEN186 | TCTTCTTCCTCCTCAGCTCG | TCTTCCTCTTCTCGCTCTCG |
| BEN189 | TTAAACCCGGTCCTGTTCTG | CATGAGCTCAGGAGGAGGAG |
| BEN200 | AAGCAAACAAACACATGCCA | TGCGGAAGAAGAAGAAAGGA |
| BEN203 | TGACTCAAATCAACCTCTCACG | TTTGTCACTCGCCATCTCTG |
| BEN206 | CGCACGCTTTATGTTCTGTC | CGTCTCCTGGTGGTACCTGT |
| BEN211 | ACACGCTCACTGGAGAATCC | TTTCGTCGGTGTAGTTGTCG |
| BEN216 | ATTGTCTTTGCTCCGACACC | TTGAGAAGCGGCGTAGAAAT |
| BEN228 | GACCAATCCTCCTCCTCCTC | TTGAAGAGCTGAGTCGGGAT |
| BEN229 | TTCAAACACGGTCTCTGCAC | GTCCAGCGATTGAAGATGGT |
| BEN25 | CGTCTACTGCCCTCAATGGT | CTAGCGAGGATTCCGATGAG |
| BEN253 | ATCTTCGTGGCTGCGTGT | GCCAACATTCGGAAAGAGAG |
| BEN269 | GAATGGGTACACGCCTGAGT | CCGATGTTGATGTTCCTGTG |
| BEN278 | AGCTGTGAGGGAGTCAAGGA | TTTCGCGGTATCTACGGAAC |
| BEN281 | GTGGATTTCTGCTCACCCAT | ATGTGAGGAGGGTGAAGGTG |
| BEN285 | ATCCGGGTTTGCACATAGAG | CCAGAGGATGTTCAGTGGGT |
| BEN288 | CAAACCGGTCACTAAACCGT | GGAAGCTGAAGAAGCACGTC |
| BEN296 | ACGCCGTGTCAGAAGAAGAT | AACAGGCTTTAACGAGGTGG |
| BEN297 | GCTCTGATGATGTTGCTCCA | CAGTTCATTAGGAGGCGAGC |
| BEN30 | GGAGAATGGTAAGCGAGAAGC | TCCACACCTTGAAAGGGTTC |
| BEN312 | TAAACCTCCGCTTAAAGCCC | ATCCGACTTCGAATTCCTCC |
| BEN316 | GCTTGCTTCTCTTGGCAATC | CCCACGGAGATAGATGGAGA |
| BEN32 | GAGATGCTTCTATCGCCTGG | CTCGTCGAGGACTCTCATCC |
| BEN327 | GCAAGATTCTTCCCGTTCAA | GCGATCTGATTAGCCCTTTG |
| BEN330 | CCTTTCTGGGGATTTGATTG | ATCCGCAGATTGGATCTTTG |
| BEN332 | CTCCGCAAGTTTCCTTTTTG | CCGACCGAGACTGGAAATTA |
| BEN335 | ATGACTCGTGATCTCCTCGG | AAATCGAGACGTTTCTCCCA |
| BEN341 | TGGAAACATTAGCTTGGAACC | GCAACTCGTCTTGTTTCATT |
| BEN343 | TCTTTGGAAGATGACCCCAG | TCCAGCTTCATCATGTCTTACA |
| BEN348 | GATTAGCTCTGGTTTCCCCC | AGAAAATCATCACCCCCTCC |
| BEN349 | GTGTGGTCGTCAATTTGTGC | AGGAGGAGGAAGAGCAGAGG |
| BEN363 | CCTTGGATTCAAAGAGCAGG | TCAGGGCGGAGTTTACAAAG |
| BEN374 | CTGACGAGGCGATTAAGAGG | TCACCATCTCAGCAGTAGCG |
| BEN376 | ATTAGGGCTGAGTCTGGGCT | GGAGCTTAACGATGTGGAACA |
| BEN387 | TGAAAGGGTCGAATTCGTTT | ATGAGTTTCGCCTAACGACG |
| BEN391 | ACCACCATGCCTAAGTCGTC | AACTAAGGTGGGGGATTTGG |
| BEN396 | GTAACAGCGTCTTGCGACAG | TCCTCTGATCCACTCCATCC |
| BEN406 | CGGGGAGGTTTTAAAGCAG | CACGGATTTGACACCCTCTT |
| BEN410 | TGTTCCTCCACCACAACAAA | GTGCTTCTGCTTCTTTTGGC |
| BEN425 | CCGGGAGTAACTGACCTCAA | TAGTCTCCTTACAACGCCCC |
| BEN43 | GTGGTGATGGTACATCTGCG | CCTTTGGTGGTAGTGGTGGT |
| BEN50 | TCAATCCTCGAATCTCGCTT | CGATCCCTGGAGGATACCTT |
| BEN52 | TTCAAGGATGAGGAAATGCC | ACAGCGAAAGGGAACAAAGA |
| BEN59 | GATTCGCTTCCCTTCCTTCT | TTGAGCTTCTCTAGCTCCGC |
| BEN6 | GTGGAAGATCGAGTGCGAAT | CCTCTGACTCCATCTCAGCC |
| BEN69 | AACCCTGAAGAATCCCGTCT | GGTTGGCTACCAGTTTCAGG |
| BEN78 | CTGTGGTTCCTGCTCTCTCC | AGTGAAATTGGGTTTCGCAC |
| BEN81 | GAAAGCGGCAAAGTCATTCT | GAATCCGAAACTCCGAGACA |
| BEN84 | TTGATCCAATAGCCATGACG | TCGAGGACGAGATGAGACCT |
| BEN98 | ATCACTTCCGGTAATGCGAC | CTGATTTGAAGGCTCTTCCG |
| BGO001 | TTCGCTATCTCTCGACCGTT | ATCCTCGCCGTGATATTCCT |
| BGO006 | AACGGCTATGGCTTGTATGG | GACTCTTCGGTCTCGCTCAG |
| BGO056 | AGGTTCGTTGGTCGGTAGTG | TTGTGGAACGAGAACTGCTG |
| BGO078 | ACTTACACCTCTTGGTGGCG | CAGCTTCTCCAAGCAGATCC |
| BGO120 | TGGCATTTGAGTAGTGGCCT | AGTGGCCCACACTTCCATAG |
| BGO146 | CCTAGTCCATGCTGTTTCGG | CCCAAGGAAGATGGTGAAGA |
| BGO153 | CGACGAAAGGAAGAAACCCT | AGAAGGAAAGTAGCCGCCTC |
| BGO156 | TGTGTCAGCCACAAGAGAGG | TCGCCATGATGTTATCTGGA |
| BGO160 | CCAGACACAACAAACATGGC | TTCGAGGAGAGTTTGGGAGA |
| BGO169 | CTAATCCACCACCGTCACCT | TTGTGTTTAGAGGCGCAATG |
| BGO179 | TTCTTCGCCTTACCTCCTGA | AACACAAGTGAGACATTGGCA |
| BGO183 | AAGAAAGGTCCATGTGTCCG | CGAAATGCCAGATTCCAAAT |
| BGO185 | GATAACCGATCGGACATTCG | AAGGCCAGTCCAACCAGAG |
| BGO196 | GTGAAGGGAAGTCCCAGACA | TCGACCACAAGACCTGTTCA |
| BGO199 | CAATCGAATCGACATGAACG | TGCGTTGTATTAGATGCACGA |
| BGR1 | TGACTTTCTTGAGGGTATGC | AGCATTGGTATCCAAGACAA |
| BGR2 | CGTACGCTTGTGTGAGTAGA | CAGCAGGTAATAACAGAGGG |
| BGR23 | GTCGGATCAGCTCTGTGTAT | CTCTTCTTCTCTCTCATCGC |
| BGR35 | CCGCCTAAGCTCATTAACTA | CACCTAGTCACCGGAATAAA |
| BGR49 | GATATTGGTCGTTGGAGTTC | CCTCTCTCGCCATTACTCTA |
| BGR52 | CAGAATCGGAGTAACCAACT | AAAATCACCGATCCTCTTCT |
| BGR55 | AGACGAAAAGGTTGCTTCTT | CTTTTAAGCTTCCTTCCTCC |
| BGR58 | AGCAGTGTATCCGCACTATC | TAATATTGTTTGGAGCAGGC |
| BGR6 | GAAGAAGCTTTACTCGGTGA | TTATGTTAGGGCTTGTCCTG |
| BGR65 | GTTGAAAGCTTACGCAGTCT | ACCTGAAGCTCTGAAACTCA |
| BGR70 | AGAGAGTTTCAGGCTTTGGT | GGGAAAAGAGTGGAGAGAGT |
| BGR71 | TCTACTTCTCTCCCTCCTCC | AGAGAGAAATGGGAGAAACC |
| BGR75 | ACACGTCTCCATGAAAAAGA | GTTCCATGGACAGAAGAAGA |
| BGR8 | ATAGTCATCGGAGGTCGATT | AAATCCACTTTCTCTCTCCC |
| BGR80 | TCGATATGTATTCAGGGGAG | GAGTCATCAGCATCTTCCTC |
| BGR83 | TCTTTCTCAGTCCCTCTTGA | GTTCCATGCAAATCCATAGT |
| BGR84 | CCTCCTTACCCTAACCCTAA | CCATAACCAGGTGAGGAAG |
| BGR87 | AGGATGGTGAAGTGAAACAG | TAATCTCCGTTGATCTCCAG |
| BGR88 | GTTCCAACCATAAGCCCT | GATAAAACCTGCGGTCAC |
| BGR93 | CAAGTCAAGTCCCAATCAAC | TTGCTGAGAGAGTGATGGA |
| BGR99 | GGATAAGCGATACACACCTT | TATGAGATACTGGTGCGTGT |
