# Supplementary material for: Identification of QTLs for Resistance to Sclerotinia Stem Rot and BnaC.IGMT5.a as a Candidate Gene of the Major Resistant QTL SRC6 in Brassica napus
Source: PLoS One. 2013 Jul 2;8(7):e67740. doi: 10.1371/journal.pone.0067740 (PMC3699613; doi:10.1371/journal.pone.0067740)
Supplement: Table S2 — Correlation coefficients of SR and LR in different environments. (DOCX) [file pone.0067740.s006.docx]

**Table S2** Correlation coefficients of SR and LR in different environments

| Trait (Locations^a^ ,Seasons) | SR (W,2009-2010) | SR (W,2010-2011) | SR (H,2010-2011) | LR (W,2010-2011) |
| --- | --- | --- | --- | --- |
| SR  (W,2010-2011) | 0.56 ^**^ |  |  |  |
| SR  (H,2010-2011) | 0.64 ^**^ | 0.52 ^**^ |  |  |
| LR  (W,2010-2011) | 0.46 ^**^ | 0.42 ^**^ | 0.18 ^**^ |  |
| LR  (W,2011-2012) | 0.42 ^**^ | 0.31 ^**^ | 0.09 | 0.48 ^**^ |

^a^ *W* Wuhan, *H* Huanggang.

*,** Significant difference at P<0.05 and P<0.01, respectively.
