# Supplementary material for: Identification of QTLs for Resistance to Sclerotinia Stem Rot and BnaC.IGMT5.a as a Candidate Gene of the Major Resistant QTL SRC6 in Brassica napus
Source: PLoS One. 2013 Jul 2;8(7):e67740. doi: 10.1371/journal.pone.0067740 (PMC3699613; doi:10.1371/journal.pone.0067740)
Supplement: Table S3 — Correlation coefficients of SR and three key growth periods (bolting, budding and flowering time) in Wuhan, 2010–2011. (DOCX) [file pone.0067740.s007.docx]

**Table S3** Correlation coefficients of SR and three key growth periods (bolting, budding and flowering time) in Wuhan, 2010-2011.

| Trait | SR |
| --- | --- |
| Bolting time | 0.11 |
| Budding time | 0.08 |
| Flowering time | 0.07 |
